# Loss of function and reduced levels of sphingolipid desaturase DEGS1 variants are both relevant in disease mechanism

**DOI:** 10.1016/j.jlr.2024.100517

**Published:** 2024-02-10

**Authors:** Michele Dei Cas, Linda Montavoci, Claudia Pasini, Anna Caretti, Sara Penati, Carla Martinelli, Umberto Gianelli, Sara Casati, Francesca Nardecchia, Annalaura Torella, Nicola Brunetti-Pierri, Marco Trinchera

**Affiliations:** 1 Department of Health Sciences, Università degli Studi di Milano, Milan, Italy; 2 S.C. di Anatomia Patologica, ASST- Santi Paolo e Carlo, Milan, Italy; 3 Department of Biomedical, Surgical and Dental Sciences, Università degli Studi di Milano, Milan, Italy; 4 Department of Human Neuroscience, Unit of Child Neurology and Psychiatry, Sapienza University of Rome, Italy; 5 Department of Precision Medicine, University of Campania "Luigi Vanvitelli", Naples, Italy; 6 Telethon Institute of Genetics and Medicine, Pozzuoli, Italy; 7 Department of Translational Medicine, Medical Genetics, University of Naples Federico II, Naples, Italy; 8 Scuola Superiore Meridionale (SSM, School of Advanced Studies), Genomics and Experimental Medicine Program, University of Naples Federico II, Naples, Italy; 9 Department of Medicine and Surgery (DMC), University of Insubria, Varese, Italy

**Keywords:** sphingolipids, ceramides, lipidomics, brain lipids, glycolipids, hypomyelinating leukodystrophy 18, dihydroceramide, sphingolipid biosynthesis, mass spectrometry

## Abstract

The last step of ex novo ceramide biosynthesis consists of the conversion of dihydroceramide into ceramide catalyzed by sphingolipid Δ4-desaturase DEGS1. DEGS1 variants were found to be responsible for heterogeneous clinical pictures belonging to the family of hypomyelinating leukodystrophies. To investigate the mechanisms making such variants pathogenic, we designed a procedure for the efficient detection of desaturase activity in vitro using LC-MS/MS and prepared a suitable cell model knocking out *DEGS1* in HEK-293T cells through CRISPR-Cas9 genome editing (KO-DES-HEK). Transfecting KO-DES-HEK cells with DEGS1 variants, we found that their transcripts were all overexpressed as much as the WT transcripts, while the levels of cognate protein were 40%–80% lower. In vitro desaturase activity was lost by many variants except L175Q and N255S, which maintain a catalytic efficiency close to 12% of the WT enzyme. Metabolic labeling of KO-DES-HEK with deuterated palmitate followed by LC-MS/MS analysis of the formed sphingolipids revealed that the ceramide/dihydroceramide and sphingomyelin/dihydrosphingomyelin ratios were low and could be reverted by the overexpression of WT DEGS1 as well as of L175Q and N255S variants, but not by the overexpression of all other variants. Similar analyses performed on fibroblasts from a patient heterozygous for the N255S variant showed very low variant DEGS1 levels and a low ratio between the same unsaturated and saturated sphingolipids formed upon metabolic labeling, notwithstanding the residual activity measured at high substrate and homogenate protein concentrations. We conclude that loss of function and reduced protein levels are both relevant in disease pathogenesis.

The biosynthesis ex novo of ceramide (Cer), glycosphingolipids, and sphingomyelin (SM) starts from acyl-CoA and serine in a pathway involving multiple steps (1). The last one is the conversion of dihydroceramide (DHCer) into Cer which is achieved through a desaturating reaction (2). Such reaction requires a desaturase enzyme activity, cytochromes, oxygen, and NADPH or NADH (2, 3, 4). The human genome encodes distinct desaturases which are able to introduce double bonds in a sphingoid base named DEGS1, DEGS2, and FADS3 (5, 6). Very recently, several DEGS1 variants were described and reported to be responsible for clinical pictures belonging to the wide family of hereditary hypomyelinating leukodystrophies (7, 8, 9, 10, 11). At present, at least 16 DEGS1 variants have been identified as the cause of syndromes with varying severity. In some cases, patients are affected very early and suffer a failure to thrive, severe neuromotor and cognitive impairment, dysmorphism, and even early death. In other cases, there is no early growth impairment and patients suffer progressive motor and cognitive regression. Intermediate cases involve patients acquiring some skills but still suffering from delayed neuromotor and cognitive development. Despite experimental evidence concerning the role of the desaturase reaction in pathophysiology (12, 13), it is not clear why DEGS1 variants are pathogenic and how they are associated with diseases of different severity. They were found to be responsible for the reduced ratio between sphingolipids and dihydrosphingolipids which is associated with oxidative stress (14, 15, 16) and, in turn, deranges multiple metabolic pathways (17, 18, 19). Very recently, DEGS1 was reported to be a mitochondria-associated endoplasmic reticulum membrane-resident and pathogenic variants were found associated with dysfunctions, including cholesterol and phospholipid metabolism, superoxide species production, and mitochondrial respiration impairment (20).

In this article, we tried to characterize several DEGS1 variants in terms of enzyme activity, expression, and metabolic activity. We first set a convenient procedure for the efficient detection of desaturase activity in vitro using LC-MS/MS and prepared a suitable cell model knocking out *DEGS1* in HEK-293T cells through CRISPR-Cas9 genome editing (KO-DES-HEK). We selected six missense variants and two nonsense variants (Table 1) and constructed mammalian expression plasmids encoding their sequences by site directed mutagenesis. Upon transfection of such plasmids into model cells, we studied desaturase activity through an in vitro assay, mRNA and protein expression through quantitative polymerase chain reaction (qPCR) and Western blotting, respectively, and the incorporation of deuterated palmitate into Cer and SM through LC-MS/MS. Next, we repeated the same evaluations in the skin fibroblasts of an affected patient carrying the N255S variant on one allele and a partial gene deletion on the other allele. Finally, we studied the effect of DEGS1 expression on mitochondrial dynamics analyzing stable transfectants through transmission electron microscopy.

**Table 1:** List of DEGS1 variants considered in the present study

cDNA variant	Protein variant	
	Three letter code	One letter code
c.320 G>A	p.(Trp107∗)	W107∗
c.337 A>G	p.(Asn113Asp)	N113D
c.395 A>G	p.(His132Arg)	H132R
c.517 C>T	p.(Arg173∗)	R173∗
c.524 T>A	p.(Leu175Gln)	L175Q
c.565 A>G	p.(Asn189Asp)	N189D
c.764 A>G	p.(Asn255Ser)	N255S
c.839 C>T	p.(Ala280Val)	A280V

## Materials and Methods

### Chemicals and reagents

Methanol, chloroform, formic acid, ammonium formate, dibutylhydroxytoluene, deuterated palmitic-d31 acid (PAd31, Sigma-Aldrich 366897), and fatty acid–free BSA (Sigma-Aldrich 126575-10GM) were at analytical grade and purchased from Sigma-Aldrich (St. Louis, MO). All aqueous solutions were prepared using purified water at a Milli-Q grade (Burlington, MA). C13-DHCerd7 (d18:0-d7/13:0, N-tridecanoyl-D-erythro-sphinganine-d7) and lipid standards were purchased from Avanti Polar (supplied by Sigma-Aldrich, St. Louis, MO).

### Plasmid DNA constructs

To obtain DEGS1 and DEGS2 expression plasmids, their cDNAs were reverse transcribed from total RNA extracted from Hep3B cells or colon mucosa (Agilent Technologies), respectively, and used as template in the presence of Phusion Taq polymerase (Thermo Fisher Scientific) and a specific primer pair (Supplemental Table S1) containing restriction sites for HindIII and XbaI. Hygromycin resistance (HygR) plasmid pcDNA3-HygR was obtained cloning HygR cDNA using the same sites and polymerase as above. HygR cDNA carrying such ends was amplified using pMYT829 as template, a generous gift of Francesca Forti (University of Milan, Italy), and the primer pair reported (Supplemental Table S1). Reaction mixtures were prepared according to the manufacturer’s protocol and submitted to the amplification programs reported (Supplemental Table S1). The obtained fragments were purified by silica gel columns (Promega), digested with HindIII and XbaI, repurified as above, and ligated to pcDNA3 vector as reported (21). Upon transformation, plasmid DNA was prepared from individual colonies, characterized by restriction enzyme digestion, and submitted to direct DNA sequencing.

pCDNA3-DEGS1 plasmid was mutated using QuikChange II-E site-directed mutagenesis kit (Agilent Technologies) and the reported primer pairs (Supplemental Table S1), designed to introduce in the sequence (GeneBank NM_003676.4) the variants reported in Table 1. WT and variants DEGS1 cDNAs were moved from pCDNA3 plasmids and subcloned in pNF21A vector as reported (21), placing the sequences in frame with the HALO tag.

### Cell manipulation

HEK-293T cells were grown and transiently transfected with a mixture of expression plasmids (either in pcDNA3 or pFN21A-HaloTag) and luciferase reporter plasmid using Fugene HD (Promega) as previously reported (22). CRISPR-Cas9 knock out of DEGS1 gene was performed using FADS7 CRISPR/Cas9 KO Plasmid (h2) (Santa Cruz Biotechnology sc-406592-KO-2) and FADS7 HDR Plasmid (h2) (Santa Cruz Biotechnology sc-406592-HDR-2) introducing cassettes expressing puromycin resistance and red fluorescent protein at the site of Cas9 cleavage. Equal amounts of each plasmid (9 μg each) were used for transfecting HEK-293T cells (1.2 × 10^6^ cells, plated the day before in a 60 mm tissue culture dish), in the presence of 5.4 μl of Fugene-HD. Two days upon transfection, cells were trypsinized, pelleted, and resuspended in culture medium supplemented with 2 μg/ml puromycin and plated in twenty 10 mm tissue culture dishes. Colonies were allowed to grow 10–14 days and observed by fluorescence microscopy. Those homogeneously red were marked, collected through cloning cylinders, and transferred into 24-wells plates for growing. Upon expansion, individual clones were screened by Western blotting using anti DEGS1 antibody (ABCAM anti-MLD antibody ab167169, 1:15,000). Stable clones expressing L175Q or N255S DEGS1 variants were obtained cotransfecting HEK-293T cells where DEGS1 was knocked out (KO-DES-HEK) with variant cDNAs and HygR cDNA. To this aim, L175Q and N255S DEGS1 variants, cloned in pcDNA3 vector, were linearized by PstI digestion and mixed with BstBI linearized pcDNA3-HygR in a 20:1 ratio. Transfected KO-DES-HEK cells were submitted to hygromycin selection (100 μg/ml), and the obtained colonies were collected by cloning cylinders, expanded, and analyzed by fluorescence microscopy using anti-DEGS1 antibody (1:2,500 dilution). Skin fibroblasts from the patient and her father were obtained for diagnostic purposes and made available for research upon written consent and approval by the local (Azienda Ospedaliera Universitaria Federico II, Naples) internal review board. They were grown as previously reported (23). Control fibroblasts of commercial origin were as reported (23). The studies in this work abide by the Declaration of Helsinki principles.

### Transmission electron microscopy

To investigate the structural and ultrastructural features of mitochondria, cells have been gently scraped from culture flasks, fixed in 2.5% glutaraldehyde/0.13 M phosphate buffer pH 7.2–7.4 for 2 h, post fixed in 1% osmium tetroxide, dehydrated through graded ethanol and propylene oxide, and embedded in epoxy resin. Several semithin sections were prepared from each sample and stained with 0.5% toluidine blue in 1% sodium borate. Ultrathin sections of 50–60 nm were counterstained with Pt-blue solution and lead citrate, to be observed in a Jeol JEM 1010 transmission electron microscope (Jeol, Tokyo, Japan). For mitochondria quantification, the Marquez simplified method was applied for each case in printed 18 × 24 cm micrographs taken at 15,000× magnification using 10 areas at a fixed 4 mm distance from one to another. Cytoplasmic surface was measured on serial 15,000× printed micrographs previously converted in digital bitmap images by image processing and analysis in Java, ImageJ program. In each image the 1 μm scale bar preset was used for system calibration. The total number of mitochondria in each image was counted and the percentage of mitochondria per area unit = 100 μm^2^ was calculated (mitochondrial density/mass). To determine the size of mitochondria, for each case, the area of mitochondria in the transverse, longitudinal, or oblique orientations was measured using ImageJ program and finally the mean area was calculated. The aspect ratio was computed as major axis/minor axis, both determined through ImageJ program.

### Sphingolipid profile

Cell samples (less than 50 μg protein) were suspended with water (100 μl) and added with internal standard [Cer 12:0, SM 12:0, hexosylceramide (HexCer) 12:0 at 1 μM concentration in methanol, 10 μl] and a methanol/chloroform mixture (850 μl, 2:1, v/v), and extracted with an oscillator thermo-mixer for 1 h (38°C, 1,000 rpm). The overwhelmed bulk of esterified fatty acids (e.g., phospholipids, acylglycerols) was hydrolyzed by alkaline methanolysis (75 μl methanolic KOH 1M, 2 h at 38°C), and then samples were neutralized by the addition of glacial acetic acid (4 μl). After centrifugation (25 min at 20,000 *g*, 4°C), the organic phase was vacuum-evaporated in a SpeedVac concentrator (SPD130DLX-230, Thermo Fisher, Waltham, MA). The residues were dissolved in 100 μl of methanol + 0.5 mg/ml dibutylhydroxytoluene and withdrawn in a glass vial after an additional centrifugation. Pure extracts (1 μl) were directly injected in LC-MS/MS (24). The samples were analyzed by a high-sensitivity LC-MS/MS consisted of QTrap 5,500 triple quadrupole linear ion trap mass spectrometer (Sciex, Darmstadt, Germany) equipped with an ESI source and coupled with an Agilent 1,200 Infinity pump ultrahigh-pressure LC system (Agilent Technologies, Palo Alto, CA). Chromatographic separation was carried out on a reverse-phase Acquity UPLC BEH C8 column 1.7 μm particle size, 100 × 2.1 mm (Waters, Franklin, MA) at 40°C using a linear gradient elution with two solvents: 0.2% formic acid and 2 mM ammonium formate in water (solvent A) and 0.2% formic acid and 1 mM ammonium formate in methanol (solvent B). Solvent A and B were 20% and 80% at 0.00 min, respectively. Solvent B was increased to 90% from 0.00 to 3.00 min, held at 90% from 3.00 to 6.00 min, then increased to 99% from 6.00 to 17.00 min, then decreased back to 80% from 17.00 to 19.00 and held at 80% until 22.00 min for re-equilibration. The flow rate was kept constant at 0.40 ml/min during the analysis. The separated analytes were detected with a triple quadrupole MS operated in multiple reaction monitoring mode via positive ESI. The analytes detected were the sphingolipids with fatty acids from 16:0–24:0 belonging to the subclasses of DHCer, Cer, SMs, dihydrosphingomyelins (DHSM), HexCer, hexosyldihydroceramides, lactosylceramides (LacCer), lactosyldihydroceramides, gangliosides GM3, globosides globotriaosylceramide, and phytoceramides. Details can be found in in Supplementary materials (Supplemental Tables S2 and S3). The sphingoid bases profile (Supplemental Table S4) was investigated with the same apparatus and the same mobile phases, above reported, except for the use of a silica based analytical column Cortecs C18 1.6 μm, 2.1 × 100 mm (Waters, MA) at 40°C with a flow of 0.20 ml/min. The chromatographic run was set as follows: Solvent B was set to 70% and increased to 85% from 0.00 to 12.0 min, held at 99% from 12.2 to 15.0 min, then decreased back to 70% until 20.00 min for re-equilibration.

### Metabolic labeling

HEK-293T or HEK-293T clones where DEGS1 was knocked out (KO-DES-HEK) were plated in 12-wells plates (0.5 × 10^6^ cells per well) and transfected as above reported, scaling down the volumes. One day after transfection, medium was replaced by fresh regular medium supplemented with 0.1 mM PAd31. To this aim, a solution containing 2 mM PAd31 and 2 mM fatty acid–free BSA in PBS was prepared according to Haynes *et al.* (25) and diluted 1:20, v/v, in the culture media (0.035 ml of the mixture PAd31/BSA both 2 mM and 0.665 ml complete DMEM). The PAd31/BSA solution was prepared dissolving PAd31 50 mM in ethanol and adding 0.040 ml of such solution to 0.96 ml of a solution prepared in PBS and containing 2 mmol of fatty acid–free BSA. Three hours upon addition of PAd31, cells were detached by trypsinization, washed twice in PBS, and processed for analytical purposes. Skin fibroblasts were metabolically labeled following an identical procedure but were plated at a density of 0.1 × 10^6^ cells per well and labeled the day after plating. Sphingolipids were extracted from cell homogenates as already reported in the previous paragraph. DHCer, Cer, SMs, and DHSMs were analyzed using a targeted approach by LC-MS/MS combining the panel of natural sphingolipids with those that had incorporated in their structure PAd31, resulting in a significant mass shift of the precursor *m/z* by +31 Da (calculated +31.1945; experimental mean + 31.1944). Since the production from PAd31 to sphingosine d31, passing through palmitoylCoA d31, appear to be limited due to deuterium kinetic isotope effects (26), we considered only fatty acid–labeled sphingolipid species that are made of physiological sphingosine backbone and deuterated palmitic acid (25, 27). The mass spectrometry conditions for palmitic acid–labeled sphingolipids are as follows: DHCer 16:0 d31 [*m/z* 571.72 > 266.28, declustering potential (DP) 80, collision energy (CE) 35 eV], Cer 16:0 d31 (*m/z* 569.71 > 264.26, DP 80, CE 35 eV), DHSM 16:0 d31 (*m/z* 736.78 > 184.06, DP 80, CE 40 eV), SM 16:0 d31 (*m/z* 734.76 > 184.06, DP 80, CE 40 eV), and phytoCer 16:0 d31 (*m/z* 587.72 > 300.3, DP 80, CE 40 eV). The dual-labeled sphingolipids (both on sphingosine and fatty acid moieties) were checked as well by using the transitions that can be found in Supplemental Table S5. Chromatographic separation was the same already adopted for the evaluation of the sphingolipid profile.

### Enzyme assay

The standard reaction mixture contained, in a final volume of 0.02 ml, 0.2 M Tris/HCl buffer pH 7, 0.2% Triton X-100, 1 mM NADPH (not found to be necessary), various concentrations of DHCer, routinely 6 μM, and various amounts of cell homogenate as the enzyme source. To prepare cell homogenates, cells collected by trypsinization were washed twice with PBS and the pellet resuspended in ice cold Tris-HCl buffer 0.1 M pH 7.5 containing 0.5% Triton X-100, at an approximate density of 10^5^ cells/μl, kept in ice 5 min and vortexed several times until the suspension appeared homogeneous. DHCer and detergent were dissolved in chloroform/methanol, 2:1 (vol/vol), placed at the bottom of the reaction tubes, and allowed to dry overnight before adding the other reaction components. Samples were incubated at 37°C for 10–30 min (30 min optimal standard condition) and then analyzed by LC-MS/MS to quantitate the amounts of deuterated Cer formed. When the enzyme source was from transfected cells, the reaction mixture included 100 μg of homogenate protein from KO-DES-HEK.

For measuring DEGS1 activity, samples were purified by addition of methanol (75 μl) and centrifugated at 12,000 *g* for 10 min. The precipitates were discarded, and pure extracts (1 μl) were directly injected in LC-MS/MS. The samples were analyzed by the same apparatus already reported. Chromatographic separation was carried out on a reverse-phase Acquity UPLC BEH C8 column 1.7 μm particle size, 100 × 2.1 mm (Waters, Franklin, MA) at 40°C using a linear gradient elution with two solvents: 0.2% formic acid and 2 mM ammonium formate in water (solvent A) and 0.2% formic acid and 1 mM ammonium formate in methanol (solvent B). Solvent A and B were 20% and 80% at 0.00 min, respectively. Solvent B was increased to 90% from 0.00 to 3.00 min, held at 90% from 3.00 to 6.00 min, then increased to 99% from 6.00 to 10.00 min, held at 99% from 10.00 to 12.00 min, and then decreased back to 80% from 12.00 to 12.10 and held at 80% until 15.00 min for re-equilibration. The flow rate was kept constant at 0.40 ml/min during the analysis. MS data were collected by multiple reaction monitoring mode via positive ESI using the following precursor ions and product ions transitions: DHCer d7 C13:0 (*m/z* 505.52 >273.3, DP 65, CE 30 eV) and Cer d7 C13:0 (*m/z* 503.46 >271.31, DP 65, CE 30 eV). Data acquisition and processing was performed using Analyst®1.7.1 and Multi-Quant®2.1.1 software (Sciex, Darmstadt, Germany), respectively.

### High-resolution MS

A subsample was also analyzed via LC coupled to high-resolution MS (LC-HRMS) that was an ExionLC™ AD system (SCIEX) connected to ZenoTOF 7600 System (SCIEX, Concord, ON, CA) equipped with Turbo V™ ion source with ESI probe to incontrovertibly confirm the chemical identity of analytes, which are not commercially available as pure analytical standards. Chromatographic and MS conditions were adapted accordingly to the analysis, taking as reference those previously described in the paragraphs above. Data were collected in both polarities (ESI+ and ESI– in separated runs) by untargeted data-dependent method disposed to record top 20 candidate ions from *m/z* 200–1500 Da. The TOF MS accumulation time was set to 100 ms, and a CE of 35 ± 15 eV was used. A 20 ms accumulation time was used for TOF MS/MS experiments.

### Reverse transcription quantitative real-time PCR

First strand cDNA was synthesized from 1 to 2 μg of total RNA by Moloney Murine Leukemia virus reverse transcriptase. Control reactions were prepared by omitting the reverse transcriptase. cDNAs (0.2–1.0 μl of first strand reactions) were amplified in a volume of 20 μl using Sybr Premix Ex Taq (Tli RNase H Plus, Takara), ROX as reference dye and StepOnePlus instrument (Applied Biosystem Life Technologies) as reported (28). Primer sequences are listed in Supplemental Table S1. Annealing temperature was 60°C. The amounts of amplified target DEGS1 cDNA were calculated as ΔCt with respect to GAPDH and reported as ΔΔCt relative to the WT transcript.

### Western blotting

For total lysate preparation, washed cell pellets were resuspended in PBS containing HALT protease inhibitor cocktail (Thermo Fisher Scientific) and then brought to RIPA buffer (50 mM Tris-HCl pH 7.4, 1% Nonidet-P40, 0.5% Na-deoxycholate, 0.1% SDS, 150 mM NaCl) containing protease inhibitor cocktail, and kept on ice with frequent vortexing for 1 h. After spinning at 12,000 *g* for 10 min at 4°C, the clean supernatant was removed and stored at −80°C. Aliquots of protein extracts (5–60 μg of protein) were separated by 12% SDS-PAGE, transferred to a nitrocellulose membrane using Trans-Blot SD Semi Dry Transfer Cell (Bio-Rad Laboratories) and blotted with rabbit polyclonal anti-DEGS1 (Abcam anti-MLD antibody ab167169, 1:15,000), or anti-HaloTag (Promega, 1:2000), or mouse monoclonal anti β-actin (Sigma, 1:5000), followed by secondary peroxidase labeled anti rabbit or anti mouse secondary antibody, respectively, according to our published protocol (22). Chemiluminescence was revealed using Alliance imaging system (Uvitec).

### Flow cytometry

Fibroblasts grown to confluency in a T25 flask were detached by trypsinization, washed twice with PBS, fixed in 3.7% formaldehyde in PBS (0.1 ml) for 10 min at room temperature while mixing, spun at 1,750 rpm 5 min at 4°C, washed with PBS, and then permeabilized with ice-cold PBS containing 1% BSA and 0.05% Triton X-100 (0.1 ml, 2 min on ice). After spinning as above, the pellet was resuspended with 0.1 ml of PBS containing 1% BSA, 0.01% Triton-X100 (PBT buffer), and rabbit anti-DEGS1 antibody (Abcam anti-MLD antibody ab167169, dilution 1:2500) and kept on ice overnight. The day after, cells were spun as above, washed twice with PBT buffer, resuspended with 0.1 ml PBT containing anti rabbit FITC-labeled secondary antibody, and kept 1 h at room temperature in the dark. After spinning as above, cells were washed twice with PBS containing 1% BSA, resuspended with PBS (0.2–0.4 ml), and analyzed by a flow cytometer.

### Statistical analyses

Statistical analyses were performed using the two-tailed, unpaired Student’s *t* test by using GraphPad Prism 8. *P* values (∗∗∗∗*P* < 0.0001, ∗∗∗*P* < 0.001, ∗∗*P* < 0.01, and ∗*P* < 0.05) were labeled in the figure, accordingly. We planned a priori the comparisons, thus we believe that could be appropriate using a two-tailed, unpaired Student’s *t* test even with analysis with more than two groups.

## Results

### In vitro desaturase assay

**Fig. 1:**
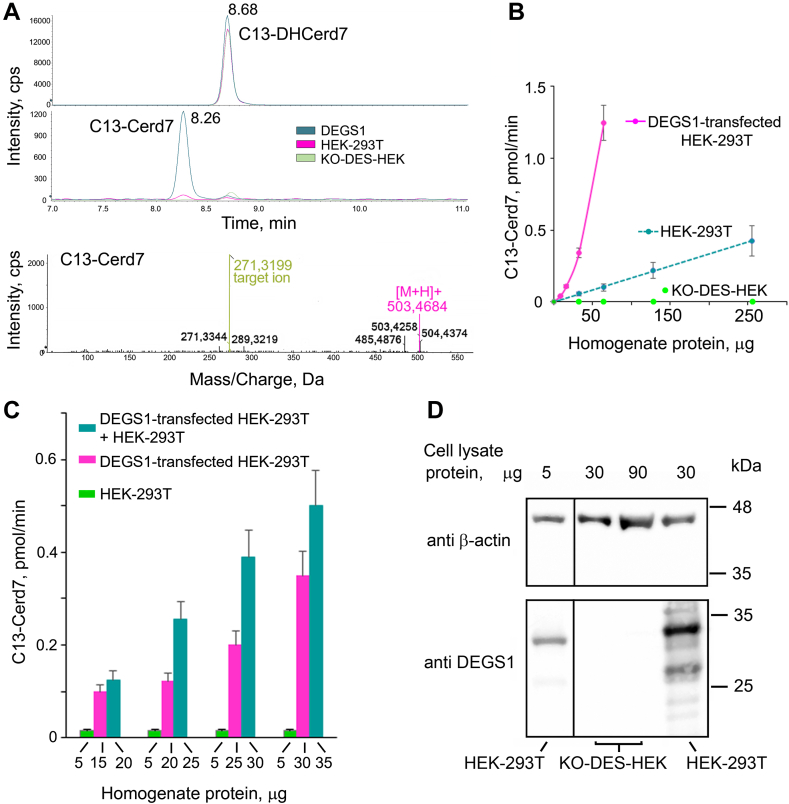
Detection of desaturase activity in vitro using C13-DHCerd7 as substrate and LC-MS/MS for C13-Cerd7 product quantitation. A: Chromatograms of C13-DHCerd7 (retention time, Rt 8.68) and C13-Cerd7 (Rt 8.26) deriving from incubation of C13-DHCer d7 (5 μM) in 30 μg of either DEGS1-transfected HEK-293T (DEGS1), mock transfected HEK-293T (HEK-293T), or HEK-293T cells where DEGS1 gene was knocked out (KO-DES-HEK). Reaction was carried out in 12 min at 37°C in 20 μl of Tris/HCl 0.1 M pH 7.5, and NADPH 1 mM. B: Various amounts of homogenate protein prepared from HEK-293T cells, DEGS1-transfected HEK-293T (transfected with pcDNA3-DEGS1), or KO-DES-HEK were incubated in reaction mixtures containing C13-DHCerd7 as substrate. Quantitation of the reaction product C13-Cerd7 was performed by LC-MS/MS. C: Various amounts of homogenate protein prepared from DEGS1-transfected HEK-293T cells were assayed alone or in the presence of a fixed amount of homogenate protein prepared from HEK-293T cells. Values are the mean ± SD of duplicate experiments performed on two independent transfections or cultured cell plates. D: Various amounts of protein extracted from HEK-293T or KO-DES-HEK were separated by 12% SDS-PAGE and transferred to a nitrocellulose membrane that was blotted with anti β-actin antibody and visualized by chemiluminescence. The membrane was then stripped and blotted again with anti DEGS1 antibody. Cer, ceramide; DHCer, dihydroceramide; KO-DES-HEK, knocking out *DEGS1* in HEK-293T cells through CRISPR-Cas9 genome editing.

We incubated C13-DHCerd7, dissolved in the presence of small amounts of Triton X-100, with homogenates obtained from HEK-293T cells or HEK-293T transfected with an expression plasmid encoding DEGS1 cDNA. By LC-MS/MS, a peak corresponding to C13-Cerd7 was found in both cases (Fig. 1A), but an 8- to 10-fold more abundant peak was detected in transfected cells, that showed about 300-fold increase in the *DEGS1* transcript by qPCR. We confirmed the chemical identity of the DEGS1 product by LC-HRMS. Using this experiment, we denoted the proper activity of the enzyme which produced from C13-DHCer d7 ([M+H]^+^ 505.5283; MS/MS 487.5234, 469.5105, 309.3501, 291.3403, 273.3292, and 214.2176) the C13-Cer d7 ([M+H]^+^ 503.4684; MS/MS 503.4258, 485.4876, 289.3219, 271.3139).

The amount of C13-Cerd7 formed by HEK-293T cells increased linearly with the amount of homogenate protein, while that formed by DEGS1 transfected cells increased almost exponentially (Fig. 1B). The concentrations and even the presence of NADPH or NADH in the reaction mixture did not affect the activity. However, the addition of homogenates from HEK-293T cells was able to make the activity measured with homogenate protein from transfected cells linear (Fig. 1C). Since cytochrome b5 and cytochrome b5 reductase were reported to be involved in the reaction (2, 4), we overexpressed them together with DEGS1 in the same transfection, but without any effect on the activity (not shown). The relevant levels of desaturase activity (Fig. 1B) and DEGS1 protein (Fig. 1D) detected in HEK-293T cells suggested that they were not suitable for the characterization of DEGS1 variants; therefore, we tried to KO endogenous DEGS1 using the CRISPR-Cas9 approach. Using commercially available plasmids targeting DEGS1 and inserting red fluorescent protein coding sequence into the endogenous gene, we obtained clones expressing red fluorescent protein that were absent of any DEGS1 expression, as proven by Western blotting, named KO-DES-HEK (Fig. 1D). Incubating C13-DHCerd7 with homogenates prepared from such clones (up to 250 μg of homogenate protein per assay), no peak corresponding to C13-Cerd7 was found (Fig. 1B), thus confirming that our assay detects the desaturase activity driven by DEGS1.

### Characterization of WT and variant DEGS1

**Fig. 2:**
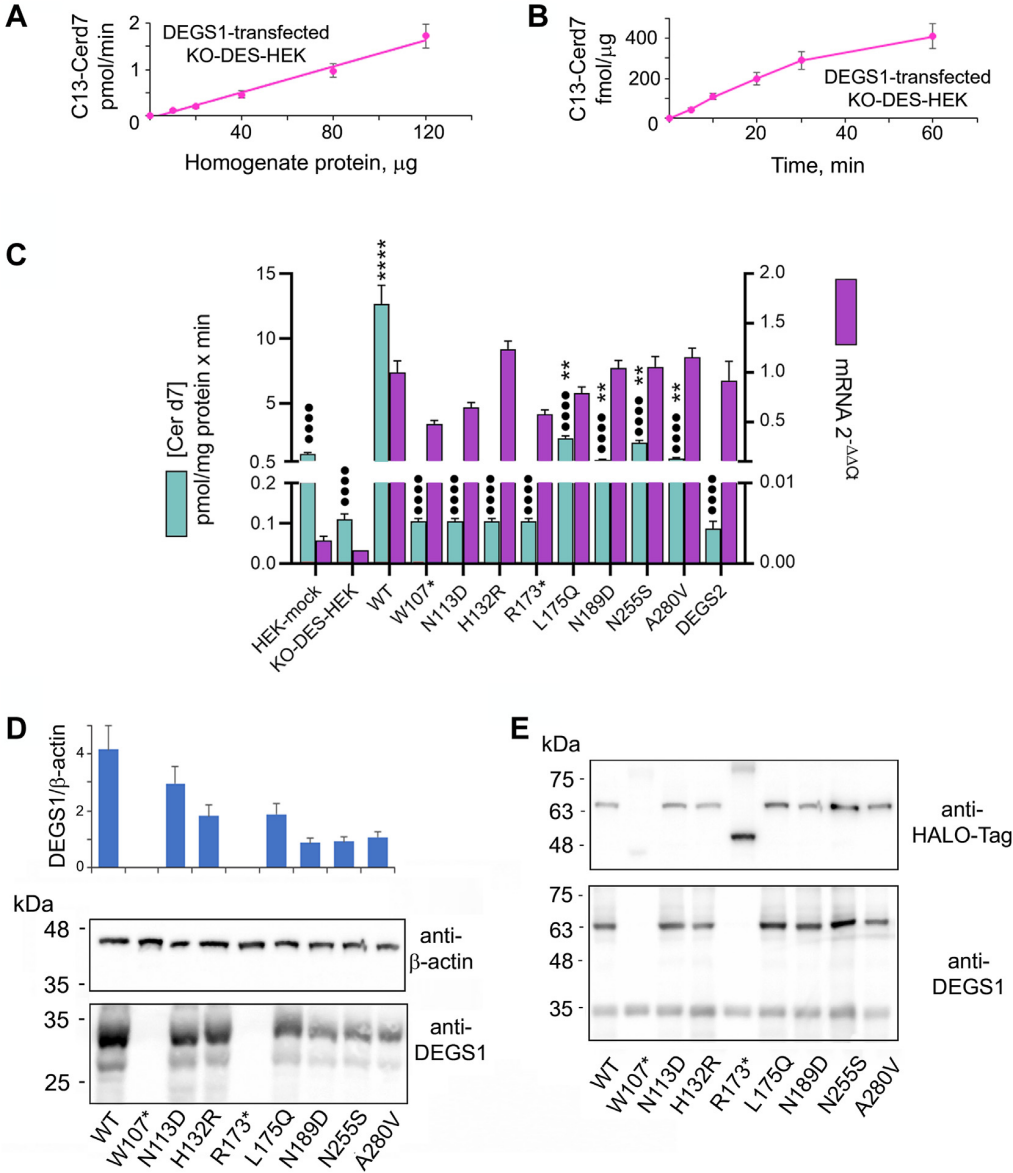
Characterization of pathogenic DEGS1 variants. A: Various amounts of homogenate protein prepared from KO-DES-HEK transfected with pcDNA3 DEGS1 (DEGS1-transfected KO-DES-HEK) were incubated in reaction mixtures containing C13-DHCerd7 as substrate. Quantitation of the reaction product C13-Cerd7 was performed by LC-MS/MS. B: Fixed amounts (80 μg) of the same homogenate protein as in panel A were incubated for different times. C: RNA and homogenate protein were prepared from mock transfected HEK-293T (HEK-mock), mock-transfected KO-DES-HEK (KO-DES-HEK mock), and KO-DES-HEK transfected with pcDNA3-based plasmids carrying WT or variant DEGS1, or DEGS2 cDNAs. RNA was reversed transcribed and individual transcripts quantitated by RT-quantitative polymerase chain reaction using GAPDH as reference housekeeping gene. Values are expressed 2^−ΔΔCt^ referred to cells transfected with WT DEGS1. Homogenate was incubated with C13-DHCerd7 for measuring desaturase activity. Significative results in the panel were investigated by *t* tests and reported as ∗ against mock transfected KO-DES-HEK and • against DEGS1 WT-transfected KO-DES-HEK. D: Five micrograms of protein extracted from KO-DES-HEK transiently transfected with pcDNA3-based plasmids carrying WT or variant DEGS1 cDNAs were separated by 12% SDS-PAGE and transferred to a nitrocellulose membrane that was blotted with anti β-actin antibody and visualized by chemiluminescence detection. The membrane was then stripped and blotted again with anti-DEGS1 antibody. Bar histogram shows the ratio between the obtained spots. Values are the mean ± SD of duplicate gels. E: Five micrograms of protein extracted from HEK-293T cells transiently transfected with pFN21-HALO-Tag based plasmids carrying WT or variant DEGS1 cDNAs was separated by 12% SDS-PAGE and transferred to a nitrocellulose membrane that was blotted with anti-DEGS1 antibody and visualized by chemiluminescence. The membrane was then stripped and blotted again with anti-HALO-Tag antibody. Cer, ceramide; DHCer, dihydroceramide; KO-DES-HEK, knocking out *DEGS1* in HEK-293T cells through CRISPR-Cas9 genome editing.

A KO-DES-HEK clone was thus transfected with pCDNA3-DEGS1 WT and the obtained homogenate used to determine optimum conditions for DEGS1 activity assay. Preliminary experiments confirmed that a linear dependence of the activity detected in transfected cells requires the addition of homogenate protein from not transfected KO-DES-HEK, and we found that 100 μg of KO-DES-HEK homogenate protein per assay is optimum to this aim. DEGS1 activity was linear up to 120 μg of homogenate protein from transfected cells (Fig. 2A) and for an incubation time of up to 60 min (Fig. 2B). KO-DES-HEK were also transfected with pcDNA3 coding various DEGS1 variants. The expression of the transcripts was assessed by RT-qPCR (Fig. 2C, purple bars) and that of the protein by Western blotting (Fig. 2D). All transcripts were strongly overexpressed (100–1,000 times over the endogenous levels) and detected at comparable levels, 0.6–1.2 times the WT transcript, with the exception of N189D variant transcript that was ten times less expressed. The amounts of DEGS1 protein expressed were not proportional to the mRNA levels. Nonsense variants W107∗ and R173∗ provided no detectable spots, while all other tested variants were detected, but at lower levels than the WT protein (Fig. 2D). By assessing the densitometry of the spots obtained from independent gels, normalized by β-actin, variants N113D, H132R, and N189D accounted for 50%–60% of WT levels and variants L175Q, N255S, and A280V for 19%–25% of WT. To evaluate the possible effect of the mutation on antibody recognition, expression plasmids carrying HALO-tagged DEGS1 variants were also transfected and the obtained homogenates analyzed by Western blot using both anti-HALO-tag and anti-DEGS1 antibodies (Fig. 2E). Only the nonsense variant W107∗ remained undetectable even by anti-HALO-tag antibodies, suggesting rapid degradation of the chimera. Conversely, only the anti-HALO-tag antibody detected the nonsense variant R173∗ as a spot of about 54 kDa, which is the predicted molecular weight of the truncated chimera. In this case, the variant appears to be expressed and no longer recognized by the anti-DEGS1 antibody. All missense variants were detected by the anti-DEGS1 antibody as much as by the anti-HALO-tag antibody.

Aliquots of the same transfected cells were also used as the enzyme source for detecting DEGS1 activity in vitro. Using the optimal reaction conditions established for WT DEGS1, all nonsense variants and the missense variants N113D and H132R appeared to be totally lacking detectable activity at any tested homogenate protein concentration. All other tested missense variants provided low but detectable activity; in particular, the L175Q and N255S variants retain residual activity in the range of 10% of WT (Fig. 2C green bars). Under such conditions, the desaturase activity measured upon transfection of DEGS2 was also faint. We also tried to detect phytoceramide upon incubation of C13-DHCer with various amounts of the same DEGS2 transfected KO-DES-HEK, but no C13-phytoCer was detectable at all.

**Fig. 3:**
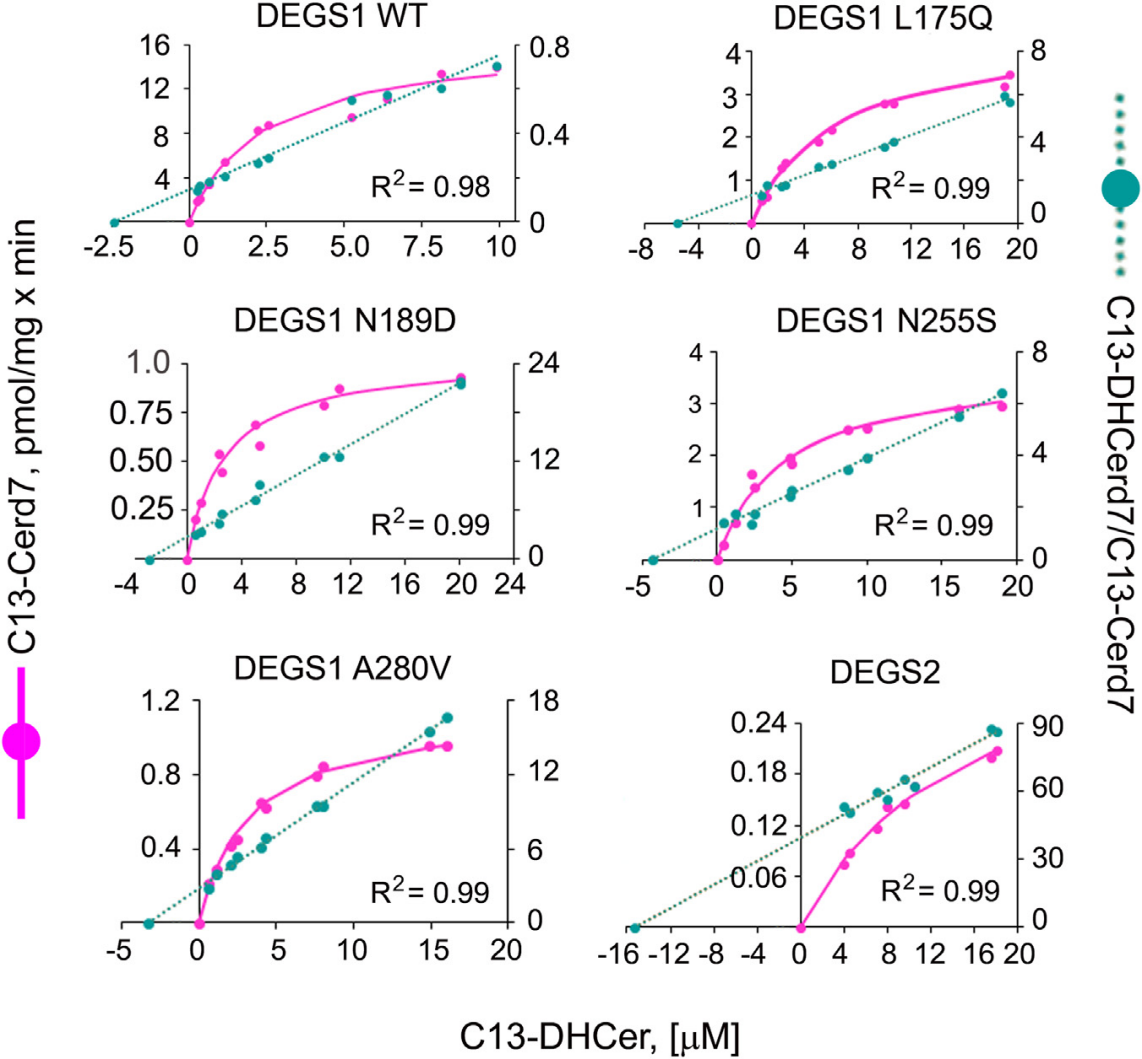
Dependence of the activity expressed in transfected KO-DES-HEK cells on the concentration of the substrate C13-DHCerd7. Hanes–Woolf plots of the activity values, presented in green glacier (secondary axis), were used for calculating kinetic constants (see Table 2). The reactions reported were obtained incubating 40 μg of WT homogenate protein for 10 min and 80 μg of DEGS1 variants and DEGS2 homogenate protein for 30 min, which assured initial rates. Linear regression was obtained via Microsoft Excel and *R*^2^ values are shown. DHCer, dihydroceramide; KO-DES-HEK, knocking out *DEGS1* in HEK-293T cells through CRISPR-Cas9 genome editing.

To better characterize desaturase activity, the kinetic properties of WT and variant DEGS1 were determined upon transfection in KO-DES-HEK (Fig. 3). The activity of WT DEGS1 was saturated at C13-DHCer concentrations over 5 μM, showing a K_m_ of 2.41 μM for the substrate. Active DEGS1 variants were all saturated at substrate concentrations higher than the WT enzyme and their Kms were greater than 2.41 μM and up to twice that in the case of N255S (Table 2). DEGS2 did not appear to be saturable at any tested substrate concentration. All variants displayed a lower V_max_ than WT, about 25% for L175Q and N255S and less than 6% for N189D and A280V. The V_max_ values of DEGS2, as well as the overall catalytic efficiency, were negligible (Table 2).

**Table 2:** Kinetic constants calculated for DEGS1, DEGS2, and DEGS1 variants upon expression in KO-DES-HEK cells. Values were obtained by the Hanes–Woolf plots presented in Fig. 3

DEGS	Vmax^a^	Km^b^	Vmax/Km
DEGS1 WT	16.5	2.41	6.85
DEGS1 L175Q	4.47	5.99	0.75
DEGS1 N189D	1.04	2.75	0.38
DEGS1 N255S	3.75	4.34	0.86
DEGS1 A280V	1.15	3.29	0.35
DEGS2	0.38	15.31	0.025

KO-DES-HEK, knocking out DEGS1 in HEK-293T cells through CRISPR-Cas9 genome editing.

a pmol/mg × min.

b μM.

### Metabolic properties of DEGS1 variants

**Fig. 4:**
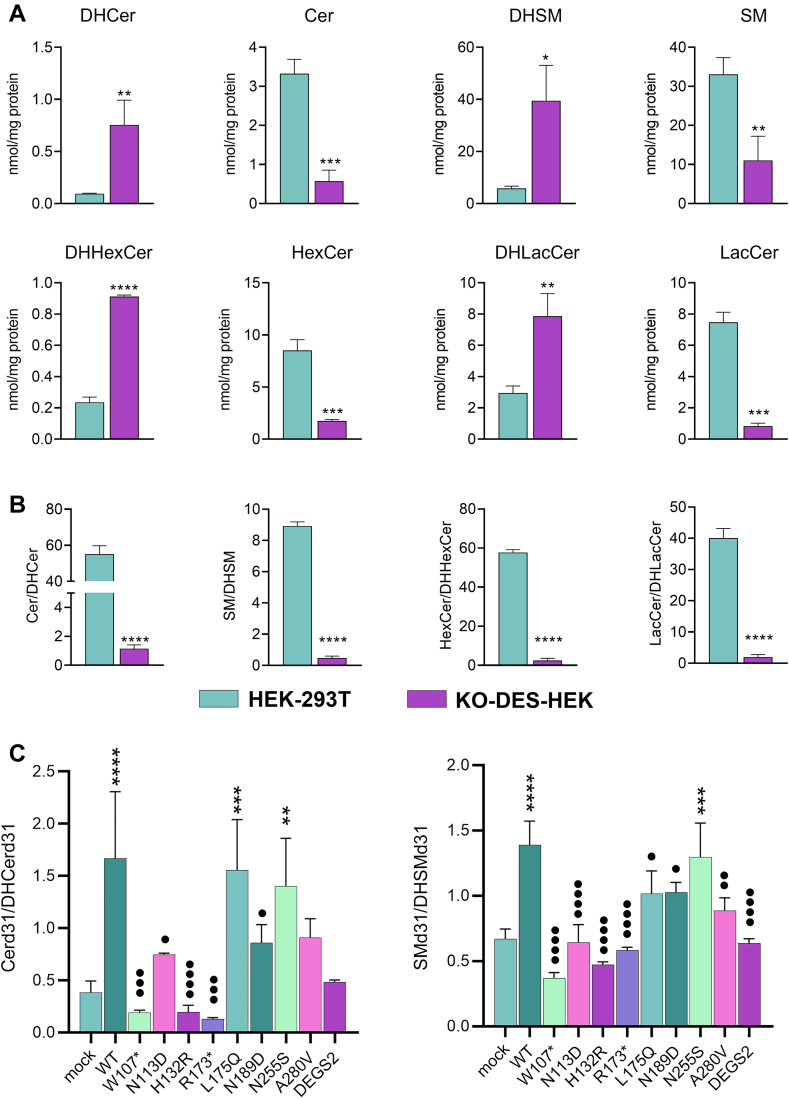
Metabolic activity of WT and variant DEGS1 in transfected cells. A: Pattern of saturated and unsaturated relevant sphingolipids detected in HEK-293T and KO-DES-HEK, as determined by LC-MS/MS. B: Ratios between individual sphingolipid and dihydrosphingolipid detected in panel A. C: Cer and SM synthesized ex novo in KO-DES-HEK upon metabolic labeling with deuterated palmitic acid (PAd31) as determined by fluxomics analysis of mock transfected KO-DES-HEK or KO-DES-HEK transfected with WT or variant DEGS1 or DEGS2. Values are reported as the ratios between Cer/DHCer and SM/DHSM. Cells were incubated for 3 h with 100 μM PAd31 to obtain labeled sphingolipids. Significant results in the panel were investigated by *t* tests and reported as ∗ against mock transfected and • against DEGS1 WT-transfected KO-DES-HEK. Cer, ceramide; DHCer, dihydroceramide; DHSM, dihydrosphingomyelin; KO-DES-HEK, knocking out *DEGS1* in HEK-293T cells through CRISPR-Cas9 genome editing; SM, sphingomyelin.

To evaluate the effect of the lack of DEGS1 activity in HEK-293T cells, we compared the sphingolipid pattern of such cells with that of KO-DES-HEK, which showed no DEGS1 activity, by LC-MS/MS. Cer, SM, HexCer, and LacCer were all decreased in KO-DES-HEK, while the corresponding saturated forms were increased (Fig. 4A). Consequently, their ratios were all significantly lowered (Fig. 4B). We also investigated the amounts of phytoCer and phytosphingosine present in the same cells and the effect of DEGS1 or DEGS2 overexpression. By LC-MS/MS analysis, we found that DEGS2 significantly affects both phytoSph and phytoCer levels, being actually able to act as hydroxylase (Supplemental Fig. S1). To investigate the ability of DEGS1 variants and DEGS2 to recover the physiological ratios, we studied the sphingolipids synthesized ex novo in transfected KO-DES-HEK upon metabolic labeling with PAd31. Before the quantification of fatty acid–labeled sphingolipids, obtained by low-resolution triple quadrupole, the metabolic products from the incubation of PAd31 in HEK-293T were investigated in LC-HRMS. In particular, we identified the sphingolipids reported in Table 3 with an accuracy of ±0.05 Da.

**Table 3:** High-resolution MS identification of palmitic acid–labeled sphingolipids in HEK-293T cells incubated for 12 h with 100 μM PAd31

Name	ESI	Q1	Q3 (MS/MS)		
DHCer 16:0 d31	+	571.7292	266.2777	284.2900	553.7086
Cer 16:0 d31	+	569.7119	264.2700	551.7000	
DHSM 16:0 d31	+	736.7870	184.0750		
SM 16:0 d31	+	734.7690	184.0735		
HexCer 16:0 d31	+	731.7670	551.6994	264.2736	
LacCer 16:0 d31	+	893.8183	551.7029	264.2734	
GM3 16:0 d31	-	1182.8859	290.0800		

DHCer, dihydroceramide; DHSM, dihydrosphingomyelin; HexCer, hexosylceramide; LacCer, lactosylceramide; PAd31, palmitic-d31 acid; Q1, molecular ion; Q3, principal daughter ions; SM, sphingomyelin.

Upon preliminary experiments, we found that a short pulse (3 h) without chase gave rise to a relevant expression of Cerd31 and SMd31 and minimal amounts of DHCerd31 and DHSMd31 in native HEK-293T cells, but the opposite effect in KO-DES-HEK, as evidenced by their ratio (Fig. 4C). The amounts of HexCer and LacCer formed were negligible under such experimental conditions and were not further considered. Moreover, due to kinetic isotope effect we verified that the unsaturated sphingolipids (Cer and SM) on dual labeled sphingolipids are 10- to 25-fold lower in respect to fatty acid–labeled sphingolipids, accounting for less than 10% of total amount of labeled sphingolipids (Supplemental Fig. S2). The transfection of KO-DES-HEK with pcDNA3 carrying the WT DEGS1 cDNA provided a substantial recovery of Cer and SM with respect to DHCer and DHSM, restoring a ratio close to that of native HEK-293T cells. Only two variants, L175Q and N255S, were found to mimic WT DEGS1 in recovering the correct Cer/DHCer and SM/DHSM ratios, while all other variants and DEGS2 failed.

### Effect of DEGS1 activity on mitochondrial structure

**Fig. 5:**
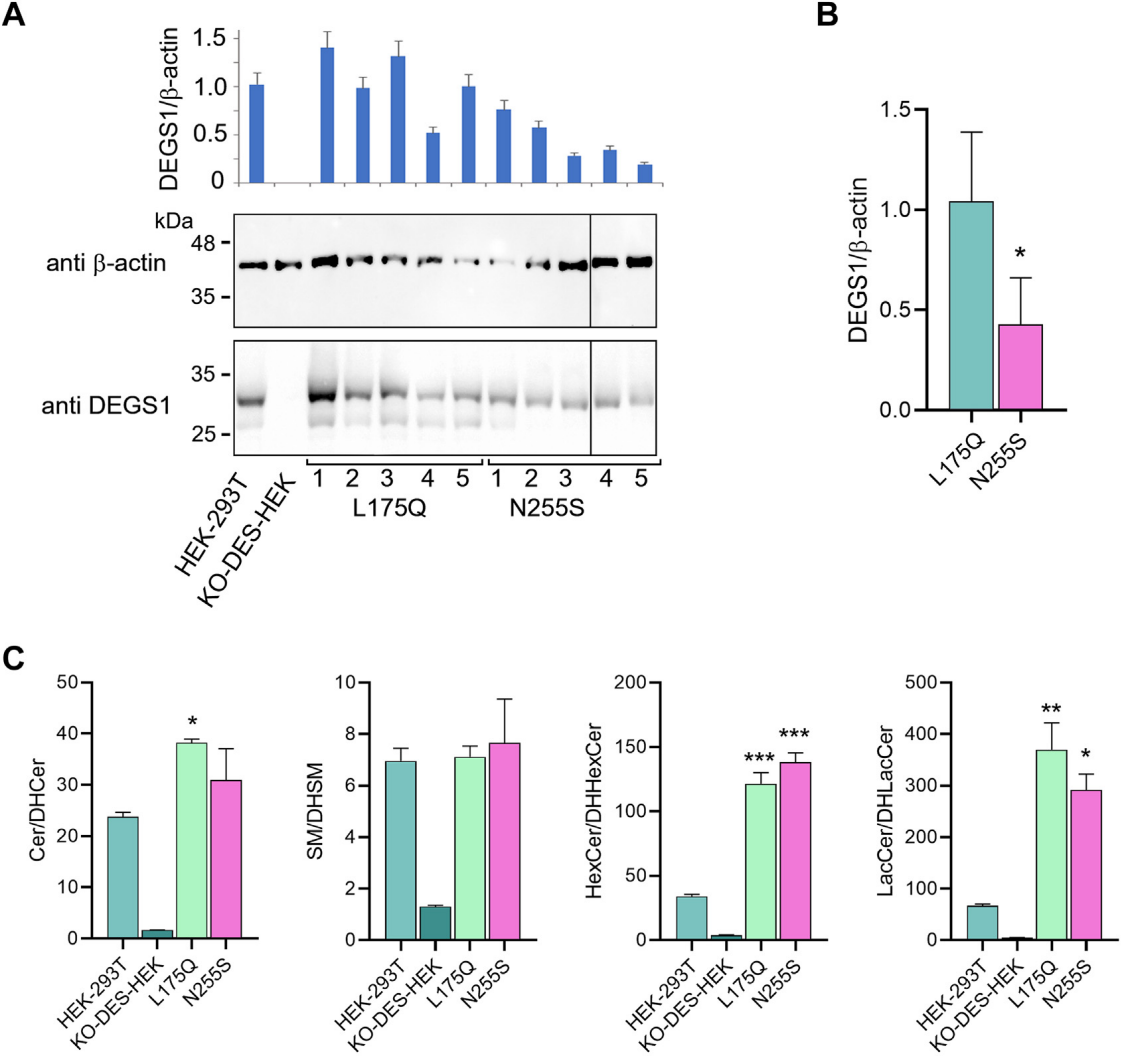
Characterization of clones permanently expressing L175Q or N255S DEGS1 variants. A: Seven micrograms of protein extracted from native HEK-293T cells, KO-DES-HEK, or five independent clones obtained from KO-DES-HEK and permanently expressing L175Q or N255S variants, respectively, were separated by 12% SDS-PAGE and transferred to a nitrocellulose membrane that was blotted with anti β-actin antibody and visualized by chemiluminescence detection. The membrane was then stripped and blotted again with anti-DEGS1 antibody. Bar histogram shows the ratio between the obtained spots. Values are the mean ± SD of duplicate gels. B: The average values of the ratios obtained for L175Q and N255S clones were compared. C: Ratios between saturated and unsaturated relevant sphingolipids detected in native HEK-293T cells, KO-DES-HEK, L175Q clone 1, and N255S clone 1 of panel A, as determined by LC-MS/MS. KO-DES-HEK, knocking out *DEGS1* in HEK-293T cells through CRISPR-Cas9 genome editing.

**Fig. 6:**
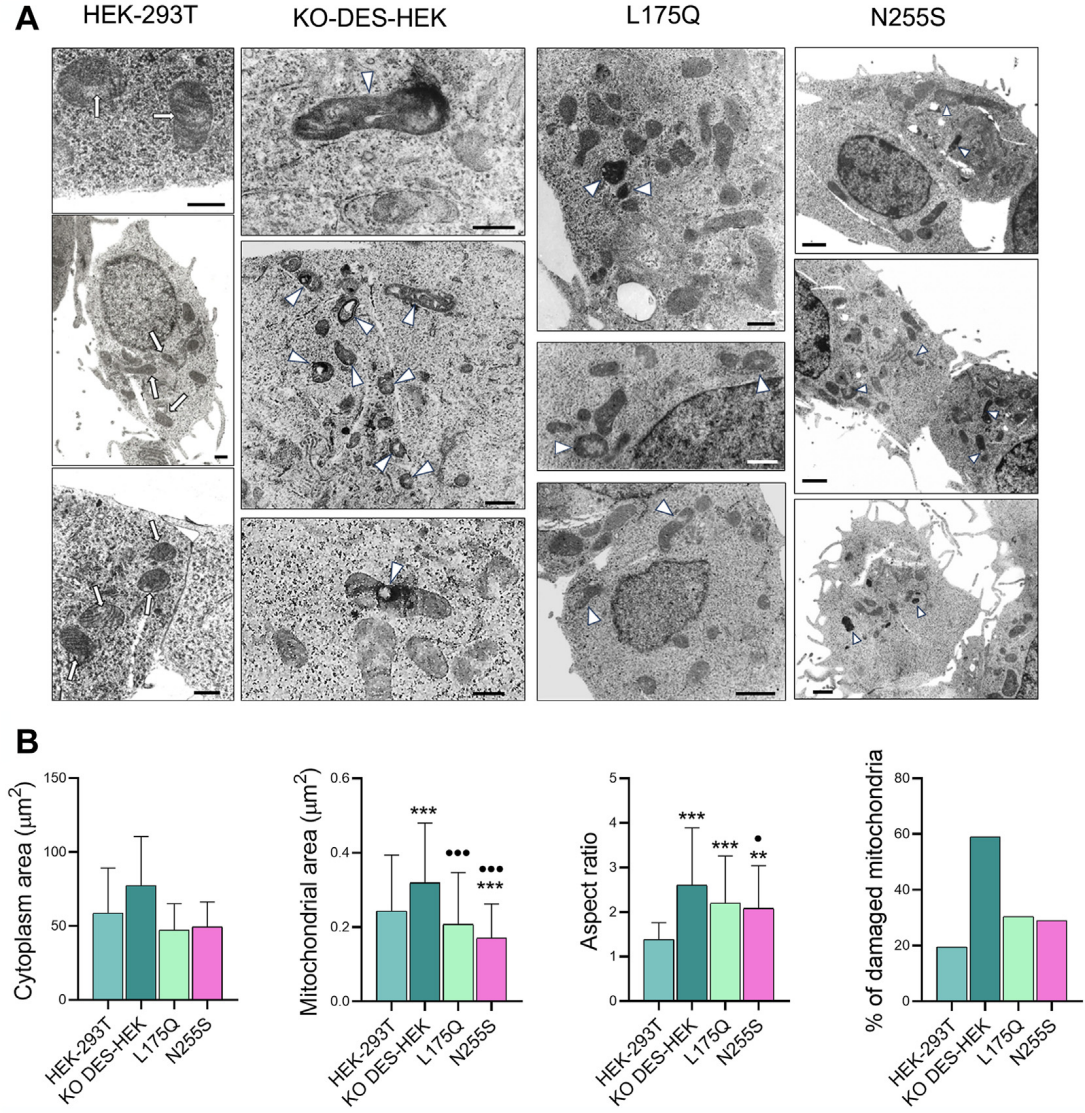
Effect of DEGS1 activity on mitochondrial structure. A: Transmission electron microscopy micrographs showing portions of cytoplasm of the cells and clones characterized in Fig. 5. Arrows indicate healthy mitochondria in native HEK-293T cells. Arrowheads indicate mitochondria with strong signs of damage in the clones, much more evident in KO-DES-HEK. Mitochondria were assumed as damaged when presenting irregular shapes (such as triangular, angular, or branched), disruption of cristae architecture, or condensed, and reduced matrix density. Micrographs are presented at different magnification and identical scale bar (500 nm). A larger view of the micrographs is available as Supplemental Figs. S3 and S4. B: Evaluation of mitochondrial damage in HEK-293T cells and clones. Micrographs taken at 15,000× magnification using 10 areas at a fixed 4 mm distance from one to another were used for mitochondria quantification and analysis (see experimental procedures). The total number of mitochondria analyzed were as follows: 113 (HEK-293T), 100 (KO-DES-HEK), 158 (L175Q clone 1), and 138 (N255S clone 1). Significative results in the panel were investigated by *t* tests and reported as ∗ against HEK-293T and • against KO-DES-HEK. The aspect ratio was computed as major axis/minor axis, both determined through ImageJ program. KO-DES-HEK, knocking out *DEGS1* in HEK-293T cells through CRISPR-Cas9 genome editing.

A recent report (20) indicated that pathogenic DEGS1 variants affect mitochondrial dynamics. To assess if our model can reproduce such functional impairment, we prepared stable clones that expressed L175Q and N255S DEGS1 variants starting from KO-DES-HEK. Five independent clones that were found to express DEGS1 homogeneously by fluorescence microscopy were analyzed by Western blotting (Fig. 5A). In the case of L175Q recombinants, DEGS1 levels were variable but similar on average to those of native HEK-293T cells. Conversely, DEGS1 protein levels were significantly lower (less than 50% on average) in N255S recombinants (Fig. 5B). The sphingolipid pattern of the clone expressing the highest levels of DEGS1 was determined by LC-MS/MS for each variant. Both L175Q and N255S were found able to restore the ratio between unsaturated and saturated Cer, SM, HexCer, and LacCer (Fig. 5C). By transmission electron microscopy, we observed a relevant increase of damaged mitochondria in KO-DES-HEK (Fig. 6A, B, Supplemental Figs. S3 and S4), about three times more than native HEK-293T cells. Mitochondria were assumed as damaged when presenting irregular shapes (such as triangular, angular, or branched), disruption of cristae architecture, or condensed and reduced matrix density. Moreover, significantly higher mitochondrial size and aspect ratio were found in KO-DES-HEK than in HEK-293T (Fig. 6B). Both L175Q and N255S clones showed a significant reduction of mitochondrial area and number of damaged mitochondria compared to KO-DES-HEK, but only N255S variant was able to significantly recover mitochondria aspect ratio (Fig. 6B).

### Desaturase activity and expression in fibroblasts from a patient carrying the N255S DEGS1 variant

**Fig. 7:**
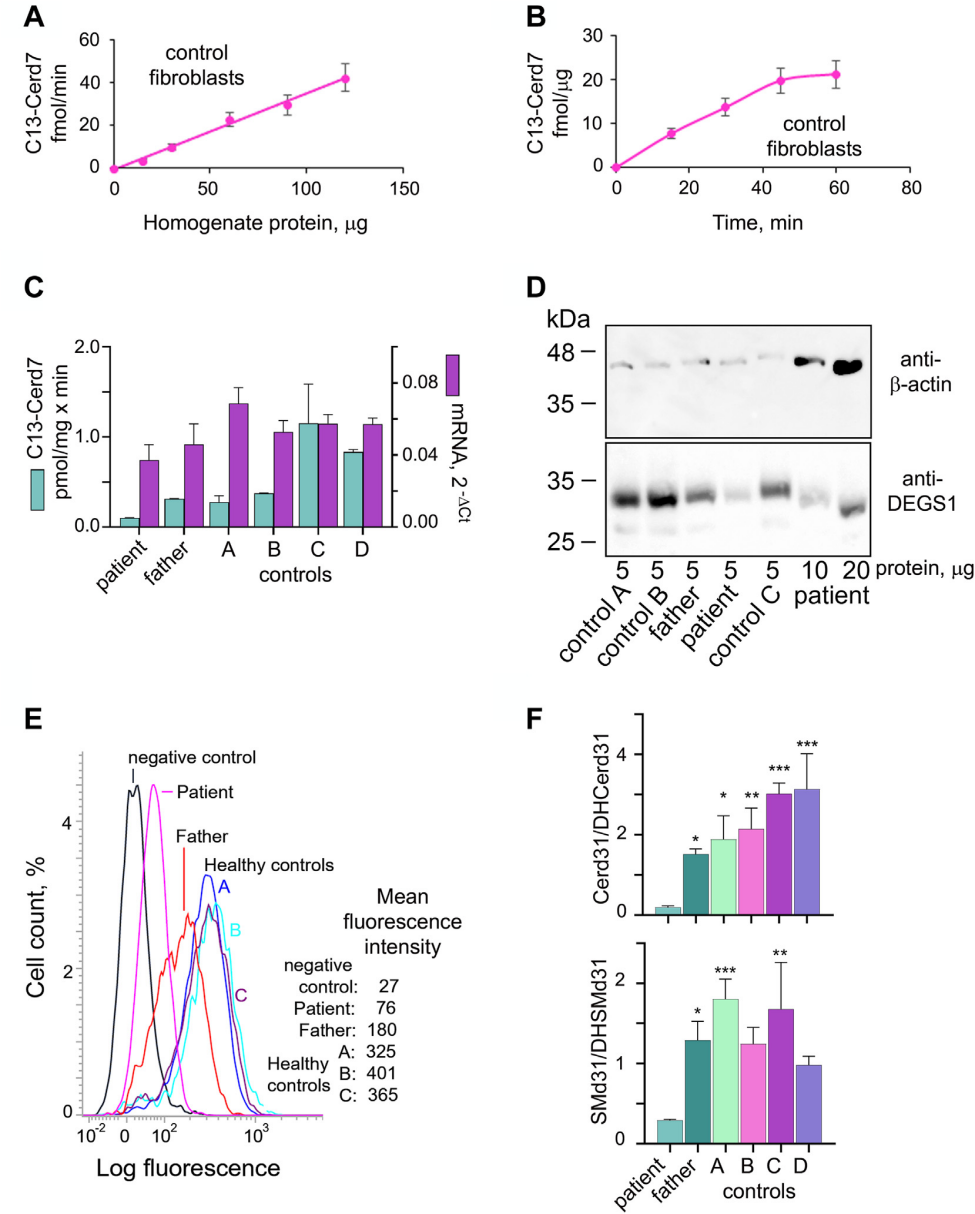
Characterization of DEGS1 variant N255S expressed in the fibroblasts of a heterozygous patient carrying a deletion in the other allele. A: Various amounts of homogenate protein prepared from normal skin fibroblasts were incubated in reaction mixtures containing C13-DHCerd7 as substrate. Quantitation of the reaction product C13-Cerd7 was performed by LC-MS/MS. B: Fixed amounts (60 μg) of the same homogenate protein as in panel A were incubated for different times. C: RNA and homogenate protein were prepared from skin fibroblasts. RNA was reversed transcribed and DEGS1 transcript quantitated by RT-quantitative polymerase chain reaction using GAPDH as reference housekeeping gene. Homogenate protein was incubated with 12 μM C13-DHCerd7 for measuring desaturase activity. *t* tests did not show significative differences. D: Protein extracted from patient and control fibroblasts was separated by 12% SDS-PAGE and transferred to a nitrocellulose membrane that was blotted with anti β-actin antibody and visualized by chemiluminescence. The membrane was then stripped and blotted again with anti-DEGS1 antibody. E: Fibroblasts were fixed, permeabilized, and stained with anti DEGS1 antibody, followed by FITC-labeled secondary antibody and analysed by flow cytometry. Negative control is control fibroblasts B stained with secondary antibody only. F: Cer and SM synthesized ex novo in fibroblasts upon metabolic labeling with deuterated palmitic acid (PAd31) were determined by fluxomics analysis. Values are reported as the ratios between Cer/DHCer and SM/DHSM. Cells were incubated with PAd31 as in Fig. 4. Tests in panels C and F were *t* tests and significative results were reported as ∗ against patient fibroblasts. Cer, ceramide; DHCer, dihydroceramide; DHSM, dihydrosphingomyelin; SM,sphingomyelin.

We studied the fibroblasts of a patient compound heterozygous for a deletion affecting two exons of *DEGS1* and the N255S variant. Fibroblasts from the healthy father, who was found to be heterozygous for the two-exon deletion in *DEGS1*, were also analyzed. DEGS1 activity was easily detected in healthy human skin fibroblasts, although at very heterogeneous levels between cells from different individuals. Such activity was linear up to 120 μg of homogenate protein and up to 45 min of incubation time (Fig. 7A, B). DEGS1 transcript levels were rather similar in patient, paternal, and control fibroblasts, while DEGS1 specific activity measured in vitro in patient fibroblasts under ideal conditions was the lowest, but not significantly lower than that measured in some controls and in paternal fibroblasts (Fig. 7C). Western blot analysis revealed very low levels of the DEGS1 protein in patient fibroblasts. It was necessary to load 20 μg of homogenate protein from patient fibroblasts to obtain a spot comparable with those obtained by loading 5 μg of homogenate protein from the father (Fig. 7D). Via flow cytometry, the mean fluorescence intensity of the DEGS1 protein in paternal fibroblasts was half of that in the control fibroblasts and only 15% in the patient’s fibroblasts (Fig. 7E).

Metabolic labeling of patient fibroblasts with PAd31 followed by LC-MS/MS analysis of the formed sphingolipids revealed that Cer/DHCer and SM/DHSM ratios were much lower than in paternal and control fibroblasts (Fig. 7F).

## Discussion

To characterize pathogenic DEGS1 variants, we have established a procedure for measuring desaturase activity in vitro not requiring radiochemicals as required in previously reported assays (2, 4, 29). Taking advantage of our recent experience with deuterated ceramide and glucosylceramide substrates (23, 30), we incubated cell homogenates with deuterated DHCer containing a C13 fatty acid as a substrate, giving rise to a deuterated C13 ceramide as a product, with molecular mass and fragmentation ions which were unambiguously distinguished by LC-MS/MS from any other endogenous compound present in the reaction mixture. As further proof that the obtained peak was the product of DEGS1 activity, we found a strong increase in the peak area in cells overexpressing the transcript and an almost complete disappearance of the peak in cells where the DEGS1 gene was knocked out. The addition of homogenate from KO-DES-HEK to the reaction was necessary to obtain a linear dependence of the activity on the amounts of protein obtained from cells overexpressing the transcript, while the concurrent overexpression of cytochrome b5 and cytochrome b5 reductase was not effective. We are unable to explain such requirement that appears related to overexpression because it was not reported using other sources (29, 31). We also found that the expression and activity of endogenous desaturase in HEK-293T cells were high enough to overlap those of transfected DEGS1 variants and thus knocked out DEGS1 by CRISPR-Cas9 genome editing, generating KO-DES-HEK where the DEGS1 protein is undetectable by Western blotting and desaturase activity is unmeasurable by in vitro assay.

KO-DES-HEK were transfected with WT DEGS1, DEGS1 variants, and DEGS2 and analyzed to determine the levels of mRNA, protein, and enzyme activity in parallel. Variant transcripts were strongly overexpressed, ruling out a potential detrimental effect of the mutation on mRNA stability and indicating a substantial reproducibility of transfection efficiency, as confirmed by luciferase assay. Conversely, the levels of overexpressed DEGS1 protein were heterogeneous, as assessed by Western blotting. The nonsense variants were both undetectable and all other missense variants appeared to be less intense than WT, particularly H132R, L175Q, N255S, and A280V. Western blots were also performed upon transfection of Halo-tagged variants and stained with both anti-Halo and anti-DEGS1 antibodies. The Halo-tagged W107∗ variant remained undetectable irrespective of the antibody used, suggesting a loss of stability, while the R173∗ variant was found at the expected size with the anti-HALO antibody only, suggesting a loss of antigenicity in this case. Halo-tagged missense variants were detected by both antibodies without differences, suggesting no loss of antigenicity in these cases. Comparing enzyme activity with mRNA and protein levels, we concluded that many variants are somewhat expressed but in a rather inactive form, while L175Q and N255S appear to be partially active and present at low levels. These in vitro data are recapitulated and confirmed by the metabolic experiment in KO-DES-HEK: unsaturated (Cer and SM) and saturated (DHCer and DHSM) sphingolipids synthesized ex novo in the cells presented an unbalanced ratio that was corrected by the overexpression of WT DEGS1 as well as of L175Q and N255S variants, but not by the overexpression of any other variant or DEGS2. Starting from KO-DES-HEK, we also prepared clones stably expressing L175Q and N255S variants, where the protein levels were closer to those expressed in native HEK-293T cells. Even in such clones, the levels of N255S variant were lower than those found in N255S or native cells but still able to rescue the proper ratio between unsaturated and saturated sphingolipids. Performing labeling experiments with deuterated palmitic acid, LC-MS/MS analysis revealed deuterated Cer and SM, but not glycosphingolipids such as glucosylceramide, lactosylceramide, globotriaosylceramide, and ganglioside GM3, which we have found to be present in both HEK-293T cells and fibroblasts analyzed by LC-MS/MS (32). We speculated that glucosylceramide synthase may have a lower affinity for ceramide containing palmitic acid, thus preferring unlabeled ceramides with longer fatty acids, as suggested by others (27).

Comparing native HEK-293T with KO-DES-HEK cells, we found a significant increase in mitochondrial damage, size, and aspect ratio in cells lacking DEGS1 activity, indicating that our model may help evaluating mitochondrial dynamics (Fig. 6A, B, Supplemental Fig. S3). In clones expressing adequate levels of L175Q or N255S variants, those retaining appreciable desaturase activity, such mitochondrial alterations are partially but significantly rescued (Fig. 6A, B, Supplemental Fig. S4), indicating the functional relevance of the residual activity measured in vitro. As shown in the recent paper reporting the link between loss of DEGS1 activity and mitochondrial dynamics (20), muscle tissues and isolated mitochondria prepared therefrom are necessary for a detailed mitochondria characterization. Our cell model, as well as patient fibroblasts (20), are only suitable for a first-level approach to mitochondria dynamics.

N255S was reported in seven patients belonging to four independent families (7, 9): in four cases (two families) as a homozygous variant and in three cases as compound heterozygous with either the N189D or L114Pfs∗11 variants. We have found that the former is inactive and the latter is also expected to be inactive because it is a truncating variant located between W107∗ and R173∗, both of which were found to be totally inactive. All four patients carrying the biallelic N255S variant presented a less severe phenotype compared to other patients (7, 9). Moreover, patients with the N255S variant on one allele showed at least acquisition of single neuromotor milestone in contrast to patients carrying biallelic truncating variants (7). We evaluated the fibroblasts of a previously unreported patient who was found to carry a heterozygous N255S variant on one allele and a likely gene-disruptive deletion on the other allele. We found that the single N255S allele is efficiently transcribed in the fibroblasts but unable to maintain the proper Cer/DHCer and SM/DHSM ratios of ex novo synthesized sphingolipids. The expression levels of DEGS1 protein were very low, as determined by both Western blot and flow cytometry. In agreement, enzyme activity was detectable only using dedicated conditions. Since the N255S variant is able to keep the Cer/DHCer and SM/DHSM ratios balanced when overexpressed or even expressed at physiological levels in KO-DES-HEK but not in patient fibroblasts, where its levels are extremely low, we concluded that the N255S variant becomes severely pathogenic only when protein levels are too low, being active enough to maintain the proper levels of unsaturated sphingolipids per se. This information may be useful for designing potential candidate therapeutics, as recently suggested for other diseases (33).

The other partially active variant, L175Q, was not found in any patient presenting symptoms of hypomyelinating leukodystrophy, or in the homozygous state, but was identified through population genetics studies and found associated with altered Cer/DHCer ratio in heterozygous healthy subjects (34). Our data suggest that this variant could be as pathogenic as N255S, confirming a previous prediction in model cells (34).

The human genome encodes a second desaturase that is potentially able to act on DHCer, DEGS2 (5); this is a bifunctional enzyme possessing hydroxylase activity toward sphingolipids (35). In HEK-293T cells the transcript levels of DEGS2 were about 1,000-fold lower than those of DEGS1. In skin fibroblasts, the DEGS2 transcript was close to the detection limit of standard qPCR and could be considered not relevant. Upon transfection in KO-DES-HEK, the DEGS2 transcript increased as much as that of DEGS1, but the activity appeared undetectable under the conditions established for DEGS1. Only by increasing DHCer concentration several times was a low activity progressively detected, suggesting a very low affinity of DEGS2 for the substrate. Upon metabolic labeling of KO-DES-HEK transfected with DEGS2, the Cer/DHCer and SM/DHSM ratios remained unbalanced, suggesting that DEGS2 is unable to rescue the loss of DEGS1, confirming a previous suggestion (7). Conversely, in DEGS2 transfected KO-DES-HEK, the amounts of phytoSph and phytoCer were significantly increased, confirming the role of DEGS2 as hydroxylase. In conclusion, we have found that many DEGS1 variants are pathogenic because they are inactive, while others, namely N255S, are active enough if maintained at proper expression levels and become pathogenic when present at low levels. Our approach appears potentially useful for the future characterization of novel DEGS1 variants.

## Data availability

All data concerned with this study are presented within this manuscript.

## Supplemental data

This article contains supplemental data.

## Conflict of interest

The authors declare that they have no conflicts of interest with the contents of this article.
