# Supplementary material for: Loss of function and reduced levels of sphingolipid desaturase DEGS1 variants are both relevant in disease mechanism
Source: J Lipid Res. 2024 Feb 10;65(3):100517. doi: 10.1016/j.jlr.2024.100517 (PMC10940770; doi:10.1016/j.jlr.2024.100517)

**Supplemental materials**

**Loss of function and reduced levels of sphingolipid desaturase DEGS1 variants are both relevant in disease mechanism**.

Michele Dei Cas^1^, Linda Montavoci^1^, Claudia Pasini^1^, Anna Caretti^1^, Sara Penati^1^, Carla Martinelli^1^, Umberto Gianelli^1,2^ Sara Casati^3^, Francesca Nardecchia^4^, Annalaura Torella^5^, Nicola Brunetti-Pierri^6,7,8^, Marco Trinchera^9^

1 Department of Health Sciences, Università degli Studi di Milano, Milan, Italy

2 S.C. di Anatomia Patologica,  ASST- Santi Paolo e Carlo, Milan, Italy

3 Department of Biomedical, Surgical and Dental Sciences, Università degli Studi di Milano, Milan, Italy

4 Department of Human Neuroscience, Unit of Child Neurology and Psychiatry, Sapienza University of Rome, Italy

5 Department of Precision Medicine, University of Campania "Luigi Vanvitelli," Naples, Italy

6 Telethon Institute of Genetics and Medicine, Pozzuoli, Italy

7 Department of Translational Medicine, Medical Genetics, University of Naples Federico II, Naples, Italy

8 Scuola Superiore Meridionale (SSM, School of Advanced Studies), Genomics and Experimental Medicine Program, University of Naples Federico II, Naples, Italy

9 Department of Medicine and Surgery (DMC), University of Insubria, Varese, Italy

**Contact information for corresponding author:**

Marco Trinchera, Dipartimento di Medicina e Chirurgia, Università dell’Insubria, via JH Dunant 5, 21100 Varese, Italy, email: marco.trinchera@uninsubria.it (to whom proofs and reprints should be addressed), tel. +39 0332 397160

**Supplemental Table S1**. Oligonucleotide primers used in the study.

Cloning primers

DEGS1 forward: 5’-CGCGAAGCTTGCGATCGCCATGGGGAGCCGCGTCTCGC

reverse: 5’-CGCGTCTAGAGTTTAAACTTACTCCAGCACCATCTCTCCTTTTTG

amplification program: 96°C 30s, [95°C 8s, 64°C 20s, 72° 45s]x30, 72°C 10 min

DEGS2 forward:5’-CGCGAAGCTTGCGATCGCCATGGGCAACAGCGCGAGCC

reverse:5’-CGCGTCTAGAGTTTAAACTCACAGACCATCTTTTGCCAGC

amplification program: 96°C 30s, [95°C 8s, 62°C 20s, 72° 45s]x30, 72°C 10 min

HygR forward: 5’ CGCGAAGCTTGCGATCGCCATGACACAAGAATCCCTGTTACTTCTC

HygR reverse: 5’ CGCGTCTAGAGTTTAAACTCAGGCGCCGGGGGCG

amplification program: 96°C 30s, [95°C 8s, 64°C 20s, 72° 45s]x30, 72°C 10 min

Forward primers: HindIII (underlined) and SgfI (double underlined) sites were added for cloning in pCDNA3 and pFN21A-HALO-Tag, respectively.

Reverse primers: XbaI (underlined) and PmeI (double underlined) sites were added for cloning in pCDNA3 and pFN21A-HALO-Tag, respectively.

Mutagenesis primers

c.320G>A forward: 5’-caaagcaatgtggaatcgct**A**gtttggaatgtttgctaatc

reverse: 5’-GATTAGCAAACATTCCAAAC**T**AGCGATTCCACATTGCTTTG

c.337A>G forward: 5’-Ctggtttggaatgtttgct**G**atcttcctattgggattcc

reverse: 5’-GGAATCCCAATAGGAAGAT**C**AGCAAACATTCCAAACCAG

c.395A>G forward: 5’-gtatcacatggatcatc**G**tcggtaccttggagctg

reverse: 5’-CAGCTCCAAGGTACCGA**C**GATGATCCATGTGATAC

c.517C>T forward: 5’-cctctcttttatgccttt**T**gacctctgttcatcaacc

reverse: 5’-GGTTGATGAACAGAGGTC**A**AAAGGCATAAAAGAGAGG

c.524T>A forward: 5’-cttttatgcctttcgacctc**A**gttcatcaaccccaaacc

reverse: 5’-GGTTTGGGGTTGATGAAC**T**GAGGTCGAAAGGCATAAAAG

c.565A>G forward: 5’-cgtatctggaagttatc**G**ataccgtggcacaggtc

reverse: 5’-GACCTGTGCCACGGTAT**C**GATAACTTCCAGATACG

c.764A>G forward: 5’-gcctctgaatttacttaccttca**G**tgtgggttatcataatgaac

reverse: 5’-GTTCATTATGATAACCCACA**C**TGAAGGTAAGTAAATTCAGAGGC

c.839C>T forward: 5’-ccactggtgaggaaaatag**T**agctgaatactatgacaacc

reverse: 5’-GGTTGTCATAGTATTCAGCT**A**CTATTTTCCTCACCAGTGG

substituted nucleotides are in boldface.

qPCR primers

DEGS1 forward: 5’-GAGATCCTGGCAAAGTATCCA

reverse: 5’-GGCAATCTCATGAATAGCCAGA

DEGS2 forward: 5’-AGATACTGGCCAAGTACCCG

reverse: 5’-GATGTCGTGGATGGCCAGC

**Supplemental Table S2.** Mass spectrometry transition for evaluation of sphingolipid profile via MRM method. MS/MS 1 is considered the quantitative fragment ion, MS/MS 2 the qualitative fragment ion, DP the declustering potential applied to molecular ion and CE the collision energy applied to molecular ion to obtain the quantitative fragment ion MS/MS 1. When chemical standards of sphingolipids were not commercially available data were also confirmed by LC-HRMS.

| **[M+H]+** | **MS/MS 1** | **MS/MS 2** | **Name** | **DP (eV)** | **CE (eV)** |
| --- | --- | --- | --- | --- | --- |
| *482.4565* | *264.2636* |  | *IS Cer 12* | *80* | *30* |
| *647.5122* | *184.0688* |  | *IS SM 12* | *80* | *40* |
| *644.5096* | *264.2636* |  | *IS GlucCer 12* | *80* | *50* |
| 540.535 | 284.2879 | 266.277 | DHCer 16 | 80 | 35 |
| 568.568 | 284.2879 | 266.277 | DHCer 18 | 80 | 35 |
| 566.5524 | 284.2879 | 266.277 | DHCer 18_1 | 80 | 35 |
| 596.6013 | 284.2879 | 266.277 | DHCer 20 | 80 | 35 |
| 624.6343 | 284.2879 | 266.277 | DHCer 22 | 80 | 35 |
| 652.6673 | 284.2879 | 266.277 | DHCer 24 | 80 | 35 |
| 650.6517 | 284.2879 | 266.277 | DHCer 24_1 | 80 | 35 |
| 538.5194 | 264.2636 | 520.51 | Cer 16 | 80 | 35 |
| 566.5524 | 264.2636 | 548.54 | Cer 18 | 80 | 35 |
| 564.5368 | 264.2636 | 546.54 | Cer 18_1 | 80 | 35 |
| 594.5857 | 264.2636 | 576.57 | Cer 20 | 80 | 35 |
| 622.6187 | 264.2636 | 604.60 | Cer 22 | 80 | 35 |
| 650.6517 | 264.2636 | 632.63 | Cer 24 | 80 | 35 |
| 648.6361 | 264.2636 | 630.62 | Cer 24_1 | 80 | 35 |
| 703.5748 | 184.0688 |  | SM 16 | 80 | 40 |
| 731.6078 | 184.0688 |  | SM 18 | 80 | 40 |
| 729.5922 | 184.0688 |  | SM 18_1 | 80 | 40 |
| 759.6411 | 184.0688 |  | SM 20 | 80 | 40 |
| 787.6741 | 184.0688 |  | SM 22 | 80 | 40 |
| 815.7071 | 184.0688 |  | SM 24 | 80 | 40 |
| 813.6915 | 184.0688 |  | SM 24_1 | 80 | 40 |
| 700.5722 | 264.2636 | 520.51 | HexCer 16 | 80 | 50 |
| 728.6052 | 264.2636 | 548.54 | HexCer 18 | 80 | 50 |
| 726.5896 | 264.2636 | 546.54 | HexCer 18_1 | 80 | 50 |
| 756.6385 | 264.2636 | 576.57 | HexCer 20 | 80 | 50 |
| 784.6715 | 264.2636 | 604.60 | HexCer 22 | 80 | 50 |
| 812.7045 | 264.2636 | 632.63 | HexCer 24 | 80 | 50 |
| 810.6889 | 264.2636 | 630.62 | HexCer 24_1 | 80 | 50 |
| 862.625 | 264.2636 | 520.51 | LacCer 16 | 80 | 60 |
| 890.658 | 264.2636 | 548.54 | LacCer 18 | 80 | 60 |
| 888.6424 | 264.2636 | 546.54 | LacCer 18_1 | 80 | 60 |
| 918.6913 | 264.2636 | 576.57 | LacCer 20 | 80 | 60 |
| 946.7243 | 264.2636 | 604.60 | LacCer 22 | 80 | 60 |
| 974.7573 | 264.2636 | 632.63 | LacCer 24 | 80 | 60 |
| 972.7417 | 264.2636 | 630.62 | LacCer 24_1 | 80 | 60 |
| 705.5948 | 184.068 |  | DHSM 16 | 80 | 60 |
| 733.6278 | 184.068 |  | DHSM 18 | 80 | 60 |
| 731.6122 | 184.068 |  | DHSM 18_1 | 80 | 60 |
| 761.6611 | 184.068 |  | DHSM 20 | 80 | 60 |
| 789.6941 | 184.068 |  | DHSM 22 | 80 | 60 |
| 817.7271 | 184.068 |  | DHSM 24 | 80 | 60 |
| 815.7115 | 184.068 |  | DHSM 24_1 | 80 | 60 |
| 702.5922 | 266.2838 | 522.511 | DHHexCer 16 | 80 | 60 |
| 730.6252 | 266.2838 | 550.541 | DHHexCer 18 | 80 | 60 |
| 728.6096 | 266.2838 | 548.541 | DHHexCer 18_1 | 80 | 60 |
| 758.6585 | 266.2838 | 578.571 | DHHexCer 20 | 80 | 60 |
| 786.6915 | 266.2838 | 606.601 | DHHexCer 22 | 80 | 60 |
| 814.7245 | 266.2838 | 634.631 | DHHexCer 24 | 80 | 60 |
| 812.7089 | 266.2838 | 632.621 | DHHexCer 24_1 | 80 | 60 |
| 864.645 | 266.2838 | 522.511 | DHLacCer 16 | 80 | 60 |
| 892.678 | 266.2838 | 550.541 | DHLacCer 18 | 80 | 60 |
| 890.6624 | 266.2838 | 548.541 | DHLacCer 18_1 | 80 | 60 |
| 920.7113 | 266.2838 | 578.571 | DHLacCer 20 | 80 | 60 |
| 948.7443 | 266.2838 | 606.601 | DHLacCer 22 | 80 | 60 |
| 976.7773 | 266.2838 | 634.631 | DHLacCer 24 | 80 | 60 |
| 974.7617 | 266.2838 | 632.621 | DHLacCer 24_1 | 80 | 60 |
| 577.3639 | 264.2636 | 292.10 | GM3 16 | 80 | 70 |
| 591.3796 | 264.2636 | 292.10 | GM3 18 | 80 | 70 |
| 590.3718 | 264.2636 | 292.10 | GM3 18_1 | 80 | 70 |
| 605.3953 | 264.2636 | 292.10 | GM3 20 | 80 | 70 |
| 619.411 | 264.2636 | 292.10 | GM3 22 | 80 | 70 |
| 633.4267 | 264.2636 | 292.10 | GM3 24 | 80 | 70 |
| 632.4189 | 264.2636 | 292.10 | GM3 24_1 | 80 | 70 |
| 1024.678 | 264.2636 | 520.51 | Gb3 16 | 80 | 70 |
| 1052.711 | 264.2636 | 548.54 | Gb3 18 | 80 | 70 |
| 1050.695 | 264.2636 | 546.54 | Gb3 18_1 | 80 | 70 |
| 1080.744 | 264.2636 | 576.57 | Gb3 20 | 80 | 70 |
| 1108.777 | 264.2636 | 604.60 | Gb3 22 | 80 | 70 |
| 1136.81 | 264.2636 | 632.63 | Gb3 24 | 80 | 70 |
| 1134.795 | 264.2636 | 630.62 | Gb3 24_1 | 80 | 70 |

**Supplemental Table S3.** Mass spectrometry transition for evaluation of phytosphingolipid (Cer t18:0) profile via MRM method.

| **[M+H]+** | **MS/MS 1** | **MS/MS 2** | **Name** | **DP (eV)** | **CE (eV)** |
| --- | --- | --- | --- | --- | --- |
| 482.7 | 264.26 |  | Cer IS | 80 | 30 |
| 644.5 | 264.26 |  | HexCer IS | 80 | 50 |
| 556.53 | 300.2909 | 282.2791 | Cer(t18:0/16:0) | 80 | 35 |
| 584.5613 | 300.2909 | 282.2791 | Cer(t18:0/18:0) | 80 | 35 |
| 612.5926 | 300.2909 | 282.2791 | Cer(t18:0/20:0) | 80 | 35 |
| 640.6239 | 300.2909 | 282.2791 | Cer(t18:0/22:0) | 80 | 35 |
| 668.6552 | 300.2909 | 282.2791 | Cer(t18:0/24:0) | 80 | 35 |
| 666.5505 | 300.2909 | 282.2791 | Cer(t18:0/24:1) | 80 | 35 |

**Supplemental Table S4.** Mass spectrometry transition for evaluation of sphingoid bases profile via MRM method.

| **[M+H]+** | **MS/MS 1** | **MS/MS 2** | **Name** | **DP (eV)** | **CE (eV)** |
| --- | --- | --- | --- | --- | --- |
| 288.4 | 252.0 |  | Sph IS | 45 | 25 |
| 300.287 | 264.3 | 282.3 | Sph | 45 | 25 |
| 302.3053 | 266.3 | 284.3 | DHSph | 45 | 25 |
| 380.256 | 264.27 | 282.3 | S1P | 45 | 25 |
| 382.4 | 284.5 | 266.3 | DHS1P | 45 | 25 |
| 318.2993 | 282.3 | 264.3 | PhytoSph | 45 | 25 |

**Supplemental Table S5.** Mass spectrometry transition for ex novo synthesized sphingolipids by incorporation of d31 palmitic acid in both the sphingosine and fatty acid backbones (dual labelled) via MRM method.

| **[M+H]+** | **MS/MS 1** | **Name** | **DP (eV)** | **CE (eV)** |
| --- | --- | --- | --- | --- |
| 602.921 | 297.494 | DHCer 16 dd31 | 80 | 35 |
| 598.9874 | 293.573 | Cer 16 dd31 | 80 | 35 |
| 764.042 | 184.06 | SM 16 dd31 | 80 | 40 |
| 767.98 | 184.06 | DHSM 16 dd31 | 80 | 40 |
| 618.9189 | 331.49 | phytoCer 16 dd31 | 80 | 40 |
| 761.040 | 293.573 | HexCer 16 dd31 | 80 | 40 |
| 923.093 | 293.57 | LacCer 16 dd31 | 80 | 40 |


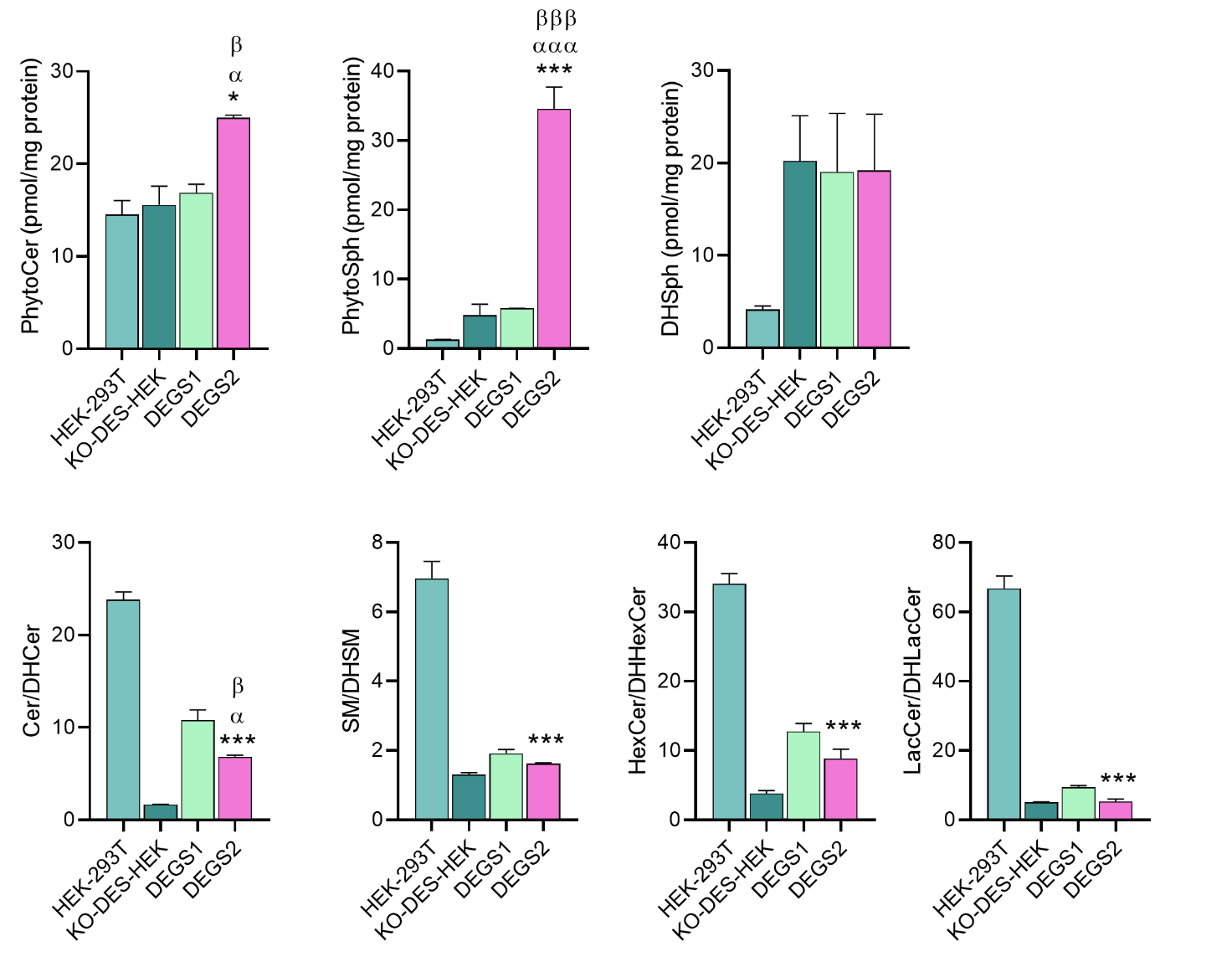


**Supplemental Figure S1.** Relevant phytosphingolipids detected in native HEK-293T cells, KO-DES-HEK, and KO-DES-HEK transfected with DEGS1 or DEGS2, as determined by LC-MS/MS. Significative results were investigated by t-tests and reported as * against HEK-293T, α against KO-DES-HEK, and ꞵ against DEGS1-transfected KO-DES-HEK.


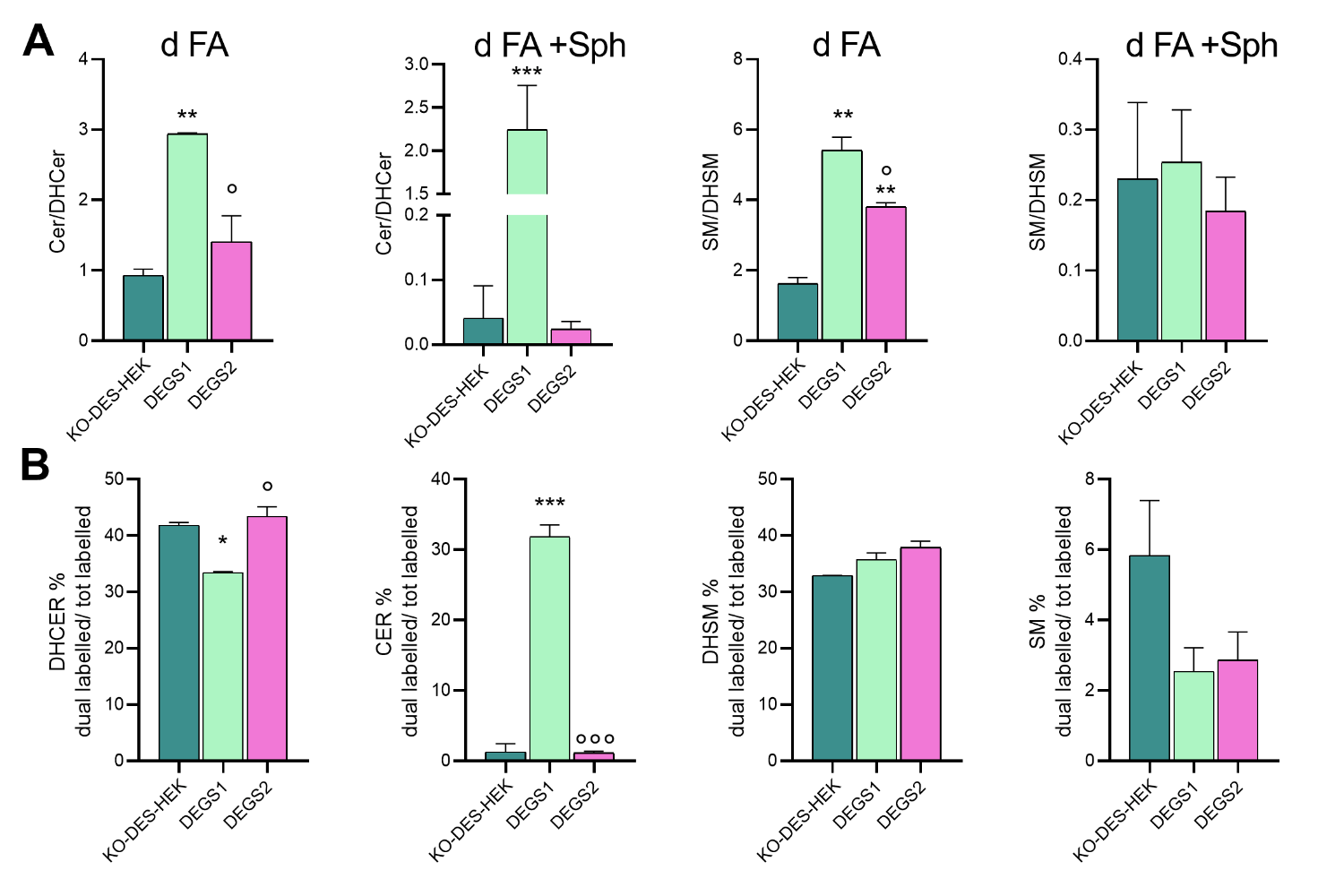


**Supplemental Figure S2.** Comparison between the ex novo synthesis of sphingolipids that have incorporated d31 palmitic acid only in the fatty acid moiety or in both the sphingosine and fatty acid backbones (dual labelled). **(A)** Ratio of unsaturated on saturated sphingolipids detected in native KO-DES-HEK, and KO-DES-HEK transfected with DEGS1 or DEGS2 as determined by LC-MS/MS. **(B)** Percentage of dual labelled sphingolipids on the total amount of labelled sphingolipids. Significative results were investigated by one way ANOVA coupled with Bonferroni post hoc test and reported as * against KO-DES-HEK whereas ^ο^ against DEGS1.


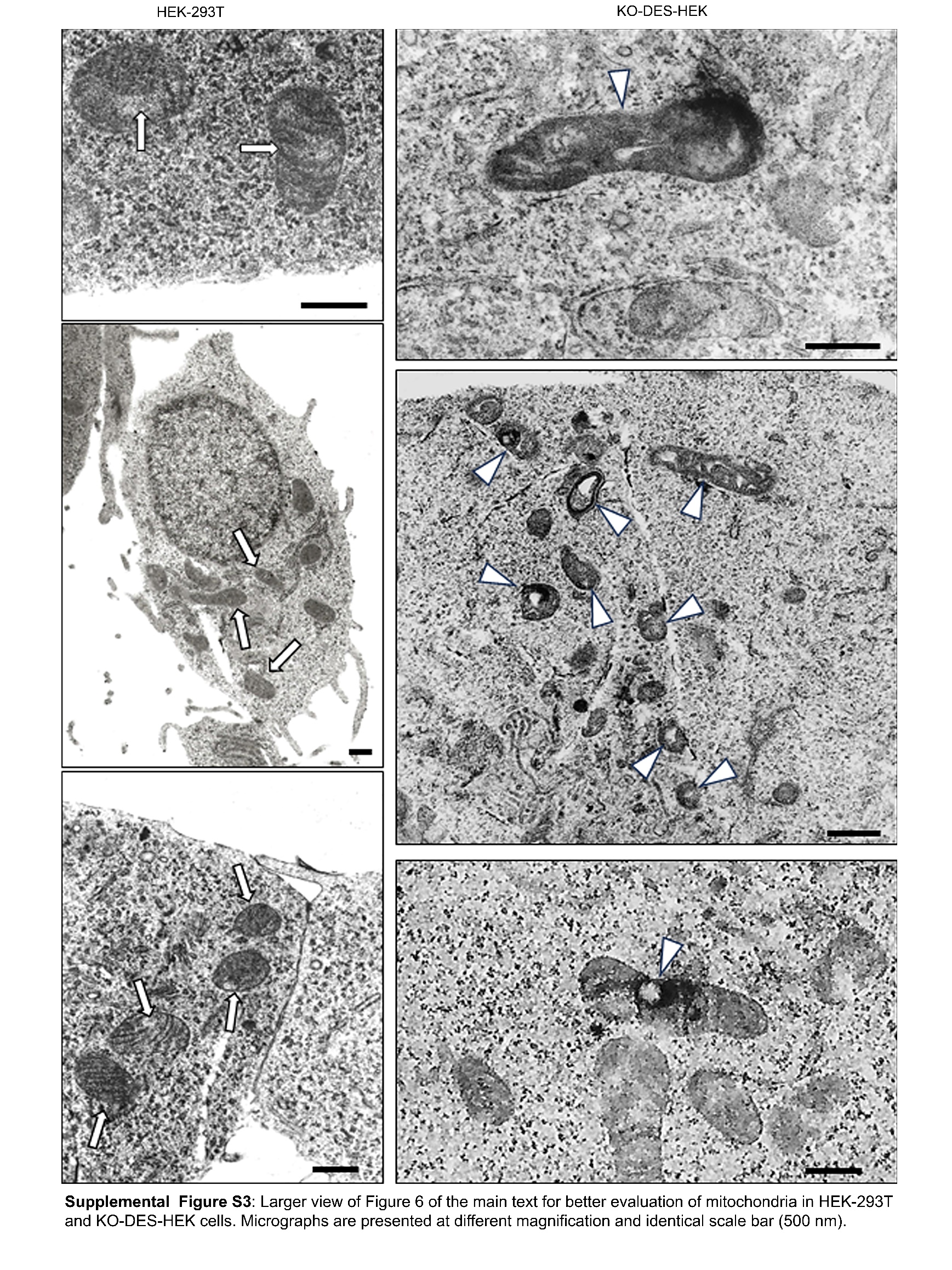


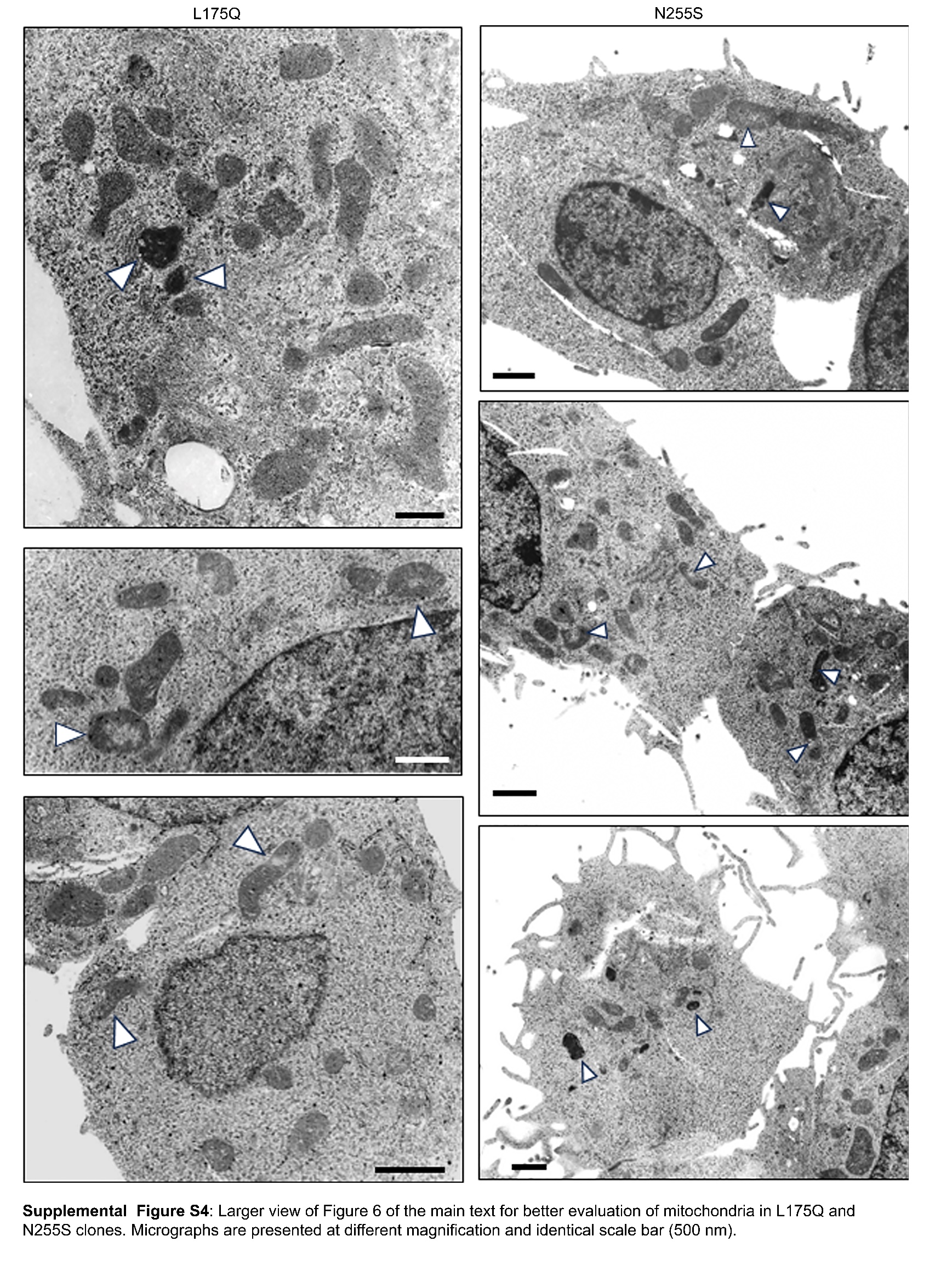

Supplement: Supplemental Data [file mmc1.docx]
